# Elevated KIF2C Expression Drives Osteosarcoma Progression by Modulating the Wnt/β‐Catenin Signaling Pathway and Contributing to an Immunosuppressive Tumor Microenvironment

**DOI:** 10.1002/cam4.70915

**Published:** 2025-04-28

**Authors:** Ya‐Yun Liu, Wu Sun, Lin Liu, Jin‐Hui Cheng, Jing‐Tang Li, Zu‐Tai Huang, Min Ouyang

**Affiliations:** ^1^ Department of Orthopaedics Jiangxi Provincial People's Hospital, the First Affiliated Hospital of Nanchang Medical College Nanchang People's Republic of China; ^2^ Discipline of Chinese and Western Integrative Medicine Jiangxi University of Chinese Medicine Nanchang People's Republic of China; ^3^ Department of Nursing Jiangxi Provincial People's Hospital, the First Affiliated Hospital of Nanchang Medical College Nanchang People's Republic of China

**Keywords:** biomarker, clinical significance, kinesin family member 2C (KIF2C), osteosarcoma

## Abstract

**Background:**

Although kinesin family member 2C (KIF2C) is implicated in various cancers, its role in osteosarcoma (OS) and the associated inflammatory microenvironment remains unclear.

**Methods:**

Publicly available datasets were analyzed to determine KIF2C expression, diagnostic value, and prognostic relevance in OS. In vitro (proliferation, colony formation, apoptosis, migration, invasion) and in vivo assays assessed its biological functions. KEGG enrichment and GSVA explored underlying pathways. ssGSEA, ESTIMATE algorithms, and single‐cell sequencing evaluated the immune context, and molecular docking and molecular dynamics identified potential inhibitory compounds.

**Results:**

KIF2C was significantly overexpressed in OS, effectively distinguishing OS from normal tissues. Elevated KIF2C levels correlated with poor survival outcomes. Silencing KIF2C suppressed OS cell proliferation, migration, invasion, and in vivo tumor growth, while promoting apoptosis; conversely, overexpression of KIF2C had the opposite effect. Mechanistically, co‐immunoprecipitation results indicated that KIF2C can bind to β‐catenin to regulate the Wnt/β‐catenin pathway. Furthermore, high KIF2C expression was associated with an immunosuppressive tumor microenvironment characterized by immune exhaustion. Molecular docking and molecular dynamics suggested butein as a candidate small‐molecule inhibitor targeting KIF2C‐related oncogenic mechanisms.

**Conclusion:**

KIF2C drives OS progression by enhancing Wnt/β‐catenin signaling and fostering an immunosuppressive microenvironment. Targeting KIF2C may offer new therapeutic approaches in managing OS.

**Abbreviations**
AUC: area under the curve; CI, Confidence interval
DEGs: differentially expressed genes
GEO: Gene Expression Omnibus
GSVA: Gene set variation analysis
KEGG: Kyoto Encyclopedia of Genes and Genomes
KIF2C: Kinesin family member 2C
OS: osteosarcoma
RGs: related genes
ROC: receiver operating characteristic
SMD: standardized mean difference
sROC: summarized receiver operating characteristic
TARGET: Therapeutically Applicable Research To Generate Effective Treatments
TCGA: The Cancer Genome Atlas database

## 1 Introduction

Osteosarcoma (OS) is among the most common primary deadly bone tumors that develop from mesenchymal tissue, accounting for roughly 20% of all primary malignant bone tumors [1]. It occurs across all age groups but is more common among teenagers. OS is characterized by rapid development, high metastatic potential, and poor clinical prognosis. Its prognosis is typically determined by tumor location and spread, surgical resection quality, and histological response to treatment. Advancements in neoadjuvant chemotherapy and surgical techniques have increased the overall survival rate over a five‐year period of patients with primary OS to around 60% of the present level [2]; nevertheless, OS remains unresponsive to standard adjuvant treatment methods such as radiotherapy and chemotherapy [3]. Over the last thirty years, the overall survival ratio of patients with OS has witnessed little improvement, necessitating a better grasp of the molecular mechanisms governing OS formation and progression, along with the quest for biomarkers and molecular treatment targets [4].

Kinesin family member 2C (KIF2C), also known as mitotic centromere‐associated kinesin, is a key regulator of mitotic spindle assembly, playing a crucial role in chromosome segregation during cell division. Located on chromosome 1p34 [5], KIF2C is essential for the correct attachment of microtubules to centromeric loci, ensuring proper chromosome alignment. Dysregulation of KIF2C can lead to abnormal chromosome segregation, resulting in aneuploidy and polyploidy, which are frequently observed in various cancers and are associated with poor prognosis [6, 7, 8]. Given the known association between KIF2C expression and aggressive tumor characteristics [9] including increased cell proliferation and metastasis, its role in cancer progression has been extensively studied in other malignancies such as glioma, colorectal cancer, and prostate cancer [10, 11, 12, 13, 14]. Recent studies have confirmed that KIF2C/MCAK plays a pivotal role in cancer progression, particularly by modulating critical cellular processes like migration, invasion, DNA repair, and immune modulation. High KIF2C expression has been linked to the hallmarks of cancer, including genome instability, resistance to cell death, activation of invasion and metastasis, immune evasion, and the avoidance of cellular senescence [15, 16]. Notably, KIF2C has been shown to enhance the malignancy of various tumors, including breast cancer, non‐small cell lung cancer (NSCLC), and hepatocellular carcinoma (HCC) [17], with elevated KIF2C expression correlating with poor overall survival and disease‐free survival [18, 19]. Additionally, KIF2C is involved in key signaling pathways, such as the Wnt/β‐catenin and mTORC1 pathways, which are crucial for cancer cell proliferation, invasion, and metastasis. In hepatocellular carcinoma, KIF2C overexpression has been linked to increased tumor cell invasion and metastasis through modulation of cytoskeletal dynamics and focal adhesion functions. These findings highlight the oncogenic potential of KIF2C across various cancer types. Given the established role of KIF2C in enhancing the malignancy of various cancers, it is reasonable to hypothesize that KIF2C may also play a significant role in osteosarcoma (OS), a highly aggressive and metastatic tumor. Although much remains to be explored regarding KIF2C's specific role in OS, its known involvement in critical cancer‐related pathways and tumor metastasis suggests that KIF2C may contribute similarly to OS progression. Osteosarcoma, characterized by its rapid progression and high metastatic potential, might share some of the carcinogenic properties associated with KIF2C expression in other cancers [20].

However, the exact role of KIF2C in OS, particularly in regulating tumor progression and metastasis, remains underexplored. Despite the critical insights gained from studying KIF2C in other malignancies, the functional and molecular mechanisms through which KIF2C influences OS progression are still poorly understood. Based on these observations, we hypothesize that elevated KIF2C expression in OS plays a crucial role in driving tumor progression. To investigate this hypothesis, we examined KIF2C expression in OS patients using public databases, revealing its upregulation and a strong association with poor prognosis. Consequently, we believe that KIF2C holds significant clinical diagnostic value in OS. Further validation through in vitro and in vivo experiments confirmed KIF2C's involvement in regulating OS cell proliferation, apoptosis, migration, and tumorigenicity. Additionally, targeted silencing of KIF2C led to a reduction in β‐catenin and cyclin D1 levels in OS cell nuclei, effectively inhibiting Wnt pathway activation. Furthermore, abnormal expression of KIF2C was found to be closely associated with the depletion of the immune microenvironment in OS. Lastly, drug prediction, molecular docking, and molecular dynamics analyses provided novel insights for the development of future molecular‐targeted therapies aimed at targeting KIF2C.

## 2 Materials and Methods

### 2.1 Compilation and Analysis Within Pan‐Cancer and OS Publicly Available Datasets

DepMap, a database for cell function screening, enables the investigation and evaluation of gene interdependence in cancer cell lines from humans [21]. This database facilitates the construction of a model of cell population dynamics measured by the Chronos score. A Chronos score of < 0 indicates a significant gene involvement in the development of the selected cell line [22]. In this study, we obtained the KIF2C CRISPR (DepMap Public 23Q2 + Score, Chronos) dataset to assess the potential role of KIF2C in malignant tumor development. To uncover the potential mechanism of KIF2C, we first classified TCGA pan‐cancer samples into two groups based on KIF2C expression: top 30% and bottom 30% [23]. Differential expression analysis was performed between these groups, and the results were used for Gene Set Enrichment Analysis (GSEA) to identify pathways potentially influenced by KIF2C across cancers. The predefined gene set used was the HALLMARK gene sets from the MSigDB database. TCGA TARGET GTEx (*N* = 19,131) in the UCSC Xena database (http://xena.ucsc.edu/) was used to explore KIF2C mRNA expression levels across various cancers [24].

We conducted a comprehensive retrieval of OS‐related sequencing and microarray datasets from the Gene Expression Omnibus and TARGET databases. We followed previously described instructions for retrieval strategy, inclusion criteria, gene annotation, and data format conversion [25]. Clinical parameters were then extracted and grouped based on information from public datasets. Herein we sequentially evaluated KIF2C expression levels in OS and normal control (NC) samples. Differences in expression levels between the groups were statistically assessed using the Wilcoxon rank‐sum test. For datasets with fewer than three groups and the same platform, we used the R package “combat” for data integration after eliminating batch effects; specifically, first, we standardized the data using the least squares method, calculating the mean and variance for each gene to make it conform to a uniform distribution. Then, we used a Bayesian statistical model to estimate the adjustment parameters for each batch. Finally, these parameters were used to adjust the original data and eliminate batch effects [26]; otherwise, the dataset was excluded. To evaluate KIF2C's total expression level in OS further, we calculated the standardized mean difference (SMD) and confidence interval (CI). Publication bias was determined using Egger and Begg tests.

To evaluate the clinical significance of KIF2C mRNA expression level in patients with OS, receiver operating characteristic (ROC) curves were constructed and AUC was calculated using the pROC package [27]. The summary ROC curve was then used to comprehensively evaluate the predictive ability of KIF2C, and the Deek test was utilized to determine publication bias. Using the Kaplan–Meier plotter database, we investigated the potential prognostic value of KIF2C in OS patients [28].

### 2.2 Immunohistochemistry (IHC)

The osteosarcoma tissue samples utilized in this study were obtained from a commercial tissue microarray provided by Guanghua Inc. (Xi'an, China), which includes tumor tissues from osteosarcoma patients and corresponding healthy bone tissue as controls. All samples were formalin‐fixed, paraffin‐embedded sections with a thickness of 4 μm. Immunohistochemical staining was conducted using an MCAK (Microtubule Depolymerase) primary antibody (Catalog No. ab70536, Abcam). Following standard procedures for deparaffinization, rehydration, and antigen retrieval, the sections were incubated with the primary antibody overnight. During antigen retrieval, tissue sections were immersed in 10 mM citrate buffer (pH 6.0) and heated at 100°C for 15 min. After natural cooling to room temperature, the sections were incubated with an HRP‐conjugated secondary antibody, followed by color development using DAB substrate. Healthy bone tissue sections were used as negative controls, stained with an irrelevant primary antibody. The antibody dilution was 1:200, with PBS buffer containing 1% BSA to block non‐specific binding. Stained sections were subsequently examined under a microscope with 20x and 40x objective lenses; using an exposure time of 500 ms, the final IHC staining score for each sample was calculated using the positive intensity and percentage scoring method [29].

### 2.3 Cell Culture

Four OS cell lines (HOS, 143B, U‐2OS, and MG63) and a human osteogenic cell line (hFOB 1.19) were obtained from the Cell Bank, Chinese Academy of Sciences (all certified). These cells were cultured in a medium containing 10% fetal bovine serum (Gibco, Thermo Fisher Scientific Inc.), 100 U/mL penicillin, and 0.1 mg/mL streptomycin (Leagene Biotechnology, Beijing, China) in a 37°C incubator (Thermo, Waltham, MA, USA) with 5% CO_2_, as previously reported [30].

### 2.4 Plasmid Construction, Transfection, and Overexpression via Plasmid

To knockdown KIF2C expression, we constructed two plasmids (sh‐KIF2C‐1,5′‐CCAACGCAGUAAUGGUUUATT‐3′ and sh‐KIF2C‐2,5′‐GCATAAGCTCCTGTGAATATA‐3′) and negative control constructs (sh‐NC) [31, 32]. According to manufacturer instructions, Lipofectamine 2000 (11668–019, Invitrogen, Carlsbad, California, USA) was utilized to transfect and subculture 143B and U‐2OS cells [31], and transfection efficiency was evaluated. To overexpress KIF2C, we first constructed an overexpression plasmid containing the full‐length KIF2C coding sequence. The KIF2C cDNA was amplified from appropriate cells using RT‐PCR and then subcloned into the pcDNA3.1 vector (Invitrogen, Carlsbad, CA, USA) [33]. The construct (pcDNA3.1‐KIF2C) was verified by DNA sequencing. 143B and U‐2OS cells were transfected with either pcDNA3.1‐KIF2C or the empty vector control using Lipofectamine 2000 according to the manufacturer's instructions. Following transfection, cells were subcultured, and the overexpression of KIF2C was confirmed by Western blot analysis.

### 2.5 Quantitative Reverse Transcription PCR (RT‐qPCR)

Total RNA was extracted from HOS, 143B, U‐2OS, and MG63 cells using a commercial total RNA extraction reagent, following the manufacturer's instructions. Briefly, 1 mL TRIzol reagent was added to the cells and mixed thoroughly, followed by the addition of 250 μL trichloromethane. After incubation for 5 min at room temperature, the mixture was centrifuged, and the resulting supernatant was transferred to a new centrifuge tube. Subsequently, 0.8 volumes of isopropanol were added, and the solution was centrifuged again. The supernatant was discarded, and the RNA pellet was washed with 1 mL of 75% ethanol before being dissolved in RNase‐free water and incubated at 55°C for 5 min. The isolated RNA was then reverse‐transcribed into cDNA using the RevertAid First Strand cDNA Synthesis Kit (Thermo, K1622) according to the manufacturer's protocol. For gene expression analysis, the synthesized cDNA was subjected to quantitative real‐time PCR (qPCR) with specific primers (KIF2C: forward 5′‐CAACCCTGCTACCGGAAGTT‐3′ and reverse 5′‐GCAGGTCAAACAGCTTCCCA‐3′; actin: forward 5′‐GAGCACAGAGCCTCGCCTTT‐3′ and reverse 5′‐TCATCATCCATGGTGAGCTGG‐3′). The PCR amplification conditions were optimized for efficient amplification and included an initial denaturation at 95°C for 15–30 s, followed by 40 cycles of denaturation at 95°C for 10 s and annealing at 60°C for 30 s. A melting curve analysis was then performed from 65°C to 105°C in one cycle to confirm the specificity of the PCR products. Gene expression levels were quantified using the 2^−ΔΔCt^ method, with actin serving as the internal reference for normalization.

### 2.6 Western Blotting

Radioimmunoprecipitation assay buffer (Beyotime, Biotechnology, Shanghai, China) containing protease and phosphatase inhibitors was used to cleave tissues and cells and extract proteins. Cell suspensions of 143B and U‐2OS cells were seeded onto coverslips in six‐well plates and incubated. As previously reported, protein samples were separated by electrophoresis, transferred onto a membrane, blocked, and incubated with primary (ab187652, Abcam, 1:1000) and secondary (GB23303, Servicebio, 1:3000) antibodies. Proteins were finally detected and visualized [25].

A commercial nuclear protein extraction kit was utilized to extract nuclear β‐catenin protein. Cells in the logarithmic growth phase were selected, and cell climbing sheets were prepared, followed by the addition of primary and secondary antibodies. Histone H3 (AF0863, 1:1000, Affinity, Changzhou, Jiangsu, China) served as the nuclear reference protein [34]. The optical density of individual bands was analyzed and quantified using ImageJ.

### 2.7 Cell Proliferation and Colony Formation Assays

#### 2.7.1 Cell Counting Kit‐8 (CCK‐8) Assay

According to manufacturer instructions, 143B and U‐2OS cells in the growth phase were digested with trypsin to obtain a single‐cell suspension. After cell counting, they were inoculated into a 96‐well plate (2 × 10^3^ cells/well), with five wells per group. The plate was then incubated at 37°C for 24, 48, 72, and 96 h. After incubation, 100 μL CCK‐8 reagent was added, and the plate was further incubated for 4 h. Optical density was then measured at 450 nm using an ELISA reader, and a growth curve was constructed.

#### 2.7.2 Colony Formation Assay

After selecting well‐growing 143B and U‐2OS cells, trypsin digestion, resuspension, and cell counting were performed. Approximately 700 cells were added to each well of a six‐well plate, followed by incubation at a constant temperature for around 2 weeks. Cell clones were subsequently fixed in 4% paraformaldehyde, stained with Giemsa, and counted [35].

### 2.8 Cell Apoptosis Assay

We collected 143B and U‐2OS cells in the logarithmic growth phase and prepared single‐cell suspensions. The cells were washed twice and the cell count was adjusted to 1 × 10^6^. A relevant working solution was added according to manufacturer instructions, and the mixture was centrifuged. The supernatant thus obtained was discarded. Cell apoptosis was assessed using the Annexin V‐FITC/PI cell apoptosis detection kit, and apoptotic cells were quantified with a FACScan flow cytometer (Becton Dickinson).

### 2.9 Cell Migration and Invasion

Scratch experiments were performed to evaluate the effects of KIF2C on the migration ability of 143B and U‐2OS cells. Stable transfections of sh‐NC, sh‐KIF2C‐1, and sh‐KIF2C‐2 cells were seeded in a six‐well plate and allowed to reach 90% confluence. The cell monolayer was then scratched using a 200 μL micropipette. The cells were washed and separated with PBS and treated with a serum‐free culture medium. The scratch width was visualized under a microscope at 0, 24, and 48 h, and quantitatively analyzed using ImageJ. The percentage of wound healing was evaluated.

To assess cell invasion, transwell invasion assays were performed in a 24‐well transwell chamber. For barrier formation, Matrigel Matrix (BD Co., cat. no. 356234, Bedford, MA, USA) was diluted with serum‐free RPMI 1640 (1:8) and added to the upper chamber for cultivation and fixation, as previously described. The cells were then counted and imaged with a high‐power microscope [36].

### 2.10 Tumor Implantations

SPF‐grade BALB/c nude mice, aged 4–6 weeks, underwent adaptive feeding for 1–2 weeks before receiving a subcutaneous injection of 0.1 mL cell suspension into their armpits. On days 1, 4, 7, 13, 19, 25, and 31, tumor length and width were measured using a vernier caliper, and tumor volume was calculated to generate a tumor growth curve. After tumor formation, on day 31 the mice were euthanized, their tumors were completely removed, and tumor weight was measured. Statistical analysis was subsequently performed on experimental data.

### 2.11 Acquisition, Enrichment Analysis, and Signature Generation of KIF2C co‐Expressed Genes Using Machine Learning‐Based Integrated Methods

Differentially expressed genes (DEGs) were identified across all datasets based on the limma and limma‐voom algorithms. We then identified genes with log_2_FC ≥ 1 and *p* < 0.05, appearing at least four times, as upregulated genes in OS. Subsequently, employing Spearman correlation analysis, we defined genes with *r* ≥ 0.3 and *p* < 0.05, appearing at least four times, as KIF2C‐relative genes. We then mapped the shared genes between these groups as KIF2C co‐expressed genes, followed by enrichment analyses.

Using the R package “clusterProfiler,” we investigated the molecular mechanisms of KIF2C co‐expressed genes in OS by KEGG pathway enrichment analysis. Further, using the R package “GOSemSim” [37], hub genes closely associated with KIF2C were screened based on molecular function and cellular component similarity [38]. To avoid potential biological significance lost in conventional differential analysis and to deeply elucidate the potential mechanism of KIF2C in OS, “hallmark gene sets” were downloaded from the “msigdbr” package. We then calculated pathway score differences between OS and NC tissues based on GSE42352, which has the most abundant sample information, to identify pathways that KIF2C may participate in or regulate in OS.

Finally, To validate the reliable consensus between KIF2C and the hub genes, we employed a comprehensive ensemble method combining seven machine learning algorithms and fourteen algorithm combinations. The GSE42352 dataset was designated as the training set, while the remaining datasets served as test sets. Each algorithm was trained by constructing multivariate logistic regression models, where the classification probabilities for each sample were computed using the logistic regression coefficients and feature expressions. In the ensemble approach, we combined the output probabilities from multiple algorithms to enhance the accuracy and stability of the classification results.

### 2.12 Evaluation of the Role of KIF2C in the Immune Microenvironment in OS

Considering that immune microenvironment dysregulation plays a crucial role in malignant tumor progression [39], we investigated the connection between KIF2C and the immune microenvironment in OS. We used the single‐sample gene set enrichment analysis (ssGSEA) method to quantify 24 immune cell infiltration levels and calculate immune scores [40]. Based on the median KIF2C expression level, we next separated the samples into groups with high and low KIF2C expression to examine differences in immune cell infiltration degrees, followed by data visualization. Furthermore, we utilized the ESTIMATE algorithm to estimate stromal and immune cell content in the OS microenvironment, assessing overall immune scores, stromal scores, tumor purity, and KIF2C expression levels [41]. A single‐cell dataset of OS from the TISCH2 database to delve deeper into potential associations between KIF2C and immune cell subsets in OS. We followed the original author's approach to perform single‐cell analysis on patient 5's sample, using quality control (QC) metrics of nFeature_RNA ≥ 300, nFeature_RNA < 4500, and percent.mt < 10 to ensure data reliability and validity. The UMAP (Uniform Manifold Approximation and Projection) algorithm was applied with a resolution of 0.5 for effective clustering of individual cells, followed by cell annotation and visualization using the SCP R package [42]. Finally, to investigate the interactions within the microenvironment, we used the CellChat R package to infer, analyze, and visualize the cell communication network [43, 44].

### 2.13 Targeted Drug Prediction, Molecular Docking, and Molecular Dynamics Simulations

As KIF2C shows promise as an OS marker, identifying potential targeted drugs for precise treatment becomes paramount. In this study, we integrated the CMap (Connectivity Map) database with the XSum algorithm to advance drug repositioning research. The CMap database is a widely used tool to identify potential drug‐disease relationships by comparing gene expression patterns [45]. It includes a large collection of chemical compounds and gene expression data across various cell lines and experimental conditions. By comparing disease gene signatures with compound signatures, CMap helps identify existing drugs that may have therapeutic effects for other diseases, thus speeding up drug repositioning. We applied the XSum algorithm to analyze and quantify the similarity between drugs and diseases [23]. The algorithm processes gene expression data by handling upregulated and downregulated genes separately, calculating cumulative expression changes in both reference and compound signatures (sumup and sumdown), and deriving a similarity score using the formula “XSum = sumup‐umdown.” Utilizing the XSum algorithm, we predicted potential small‐molecule drugs targeting KIF2C based on the Cmap database. Specifically, we divided the samples into groups according to KIF2C's median expression level, subjected these groups to log2FC differential analysis, and extracted the top 300 genes with the most significant changes to delineate OS molecular characteristics linked to KIF2C. Subsequently, we derived the CMap drug expression matrix and computed the CMap score using the XSum algorithm, with a lower score signifying a higher potential for the drug to reverse OS [46].

To identify potential targeted drugs, we performed molecular docking for further validation. We screened and downloaded the KIF2C crystal structure, determined by X‐ray diffraction, from PDB (http://www.rcsb.org/pdb/home/home.do), opting for the group with the highest resolution. Simultaneously, we identified the two‐dimensional structure of potential small‐molecule drugs through the PubChem database (https://pubchem.ncbi.nlm.nih.gov/). Subsequently, we utilized PyMOL to eliminate non‐specific chains and solvent molecules. All protein and molecular files were converted into PDBQT format, excluding water molecules and adding polar hydrogen atoms. Molecular docking studies were performed using AutoDock Vina 1.2.2 (http://autodock.scripps.edu/). We considered the docking model with Vina SCOR ≤ −4 kcal/mol as an ideal docking model, which was then visualized [47]. After that the 100 ns molecular dynamics (MD) simulations of the complexes were performed using Gromacs 2022 software. The Charmm 36 force field was applied to model the protein, while Gaff2 was used for the ligand [48]. The TIP3P water model was selected to solvate the protein‐ligand system, creating a water box with a periodic boundary of 1.2 nm [49]. Electrostatic interactions were treated using the Particle Mesh Ewald (PME) method, and the Verlet algorithm was applied for the calculations. The system was first equilibrated with 100,000 steps of isothermal‐isovolumetric ensemble (NVT) and isothermal‐isobaric ensemble (NPT) simulations, with a coupling constant of 0.1 ps and a duration of 100 ps. Both van der Waals and Coulomb interactions were calculated with a 1.0 nm cutoff. Finally, the system was simulated at constant temperature (310 K) and pressure (1 bar) for 100 ns using Gromacs 2022.

### 2.14 Co‐Immunoprecipitation (CO‐IP) Assays

First, the cells are washed with ice‐cold PBS buffer to remove the culture medium and extracellular contaminants, followed by cell lysis using RIPA buffer containing protease inhibitors to extract total protein. Next, the cell lysate is mixed with either KIF2C or β‐Catenin specific antibodies and incubated overnight at 4°C to promote binding between the antibody and the target protein. Protein A/G agarose beads are then added to capture the immune complexes, followed by incubation for 1–2 h and multiple washes to remove non‐specifically bound proteins. Finally, protein samples are separated by SDS‐PAGE gel electrophoresis, transferred to a membrane, and detected by Western blot to analyze the interaction between KIF2C and β‐Catenin. IgG antibody is used as a negative control to verify the specificity of the experiment.

### 2.15 Statistical Analysis

Wilcoxon rank‐sum test and SMD were employed to assess differences in KIF2C expression levels between the OS and NC groups. For SMD, heterogeneity was assessed using the Cochran *Q*‐test and *I* ^2^; if *I* ^2^ > 50% and chi‐squared test *p* < 0.05, a random effects model was used for evaluation; otherwise, a fixed effects model was used [50]. To assess publication bias, the Begg, Egger, and Deek's tests were employed; publication bias in SMD results was considered significant if the *p*‐value was less than 0.05. StataSE‐15 (64 bit) was used to plot summary ROC curves, and R (v4.1.0) and Prism 8 (64 bit) were employed for statistical analysis and visualization. *p* < 0.05 was deemed statistically significant unless otherwise noted.

## 3 Results

### 3.1 Exploring KIF2C Expression Level in Cancers and Clinical Significance in OS Based on Public Datasets

**FIGURE 1:**
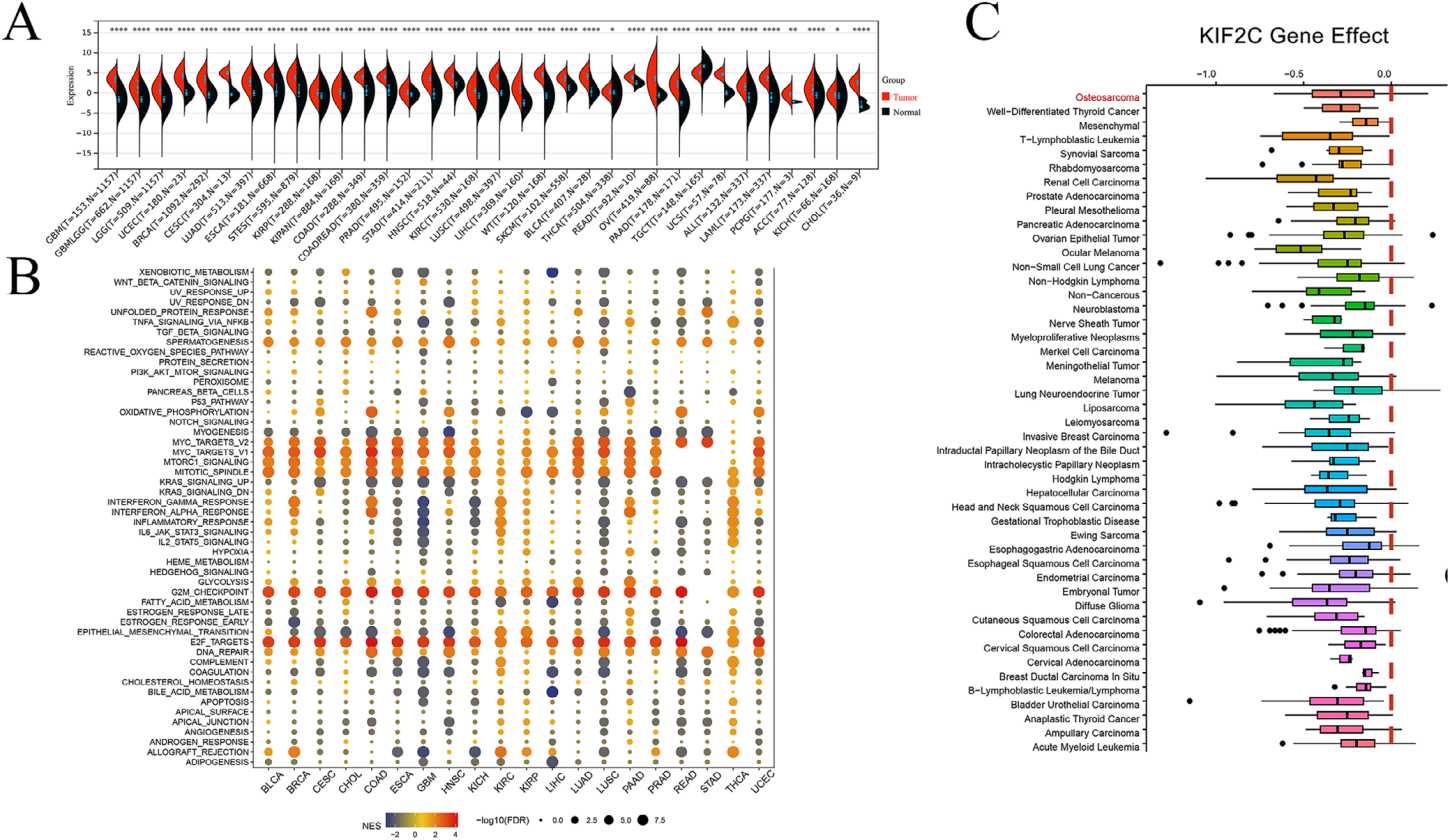
The characteristics of KIF2C in cancers. (A) The mRNA expression level of KIF2C in different types of malignant tumors. (B) GSEA regarding KIF2C expression in pancancer; NES represents enrichment score. (C) Identification of important functions of KIF2C in various malignancies, including OS. KIF2C, Kinesin Family Member 2C; OS, osteosarcoma.

**FIGURE 2:**
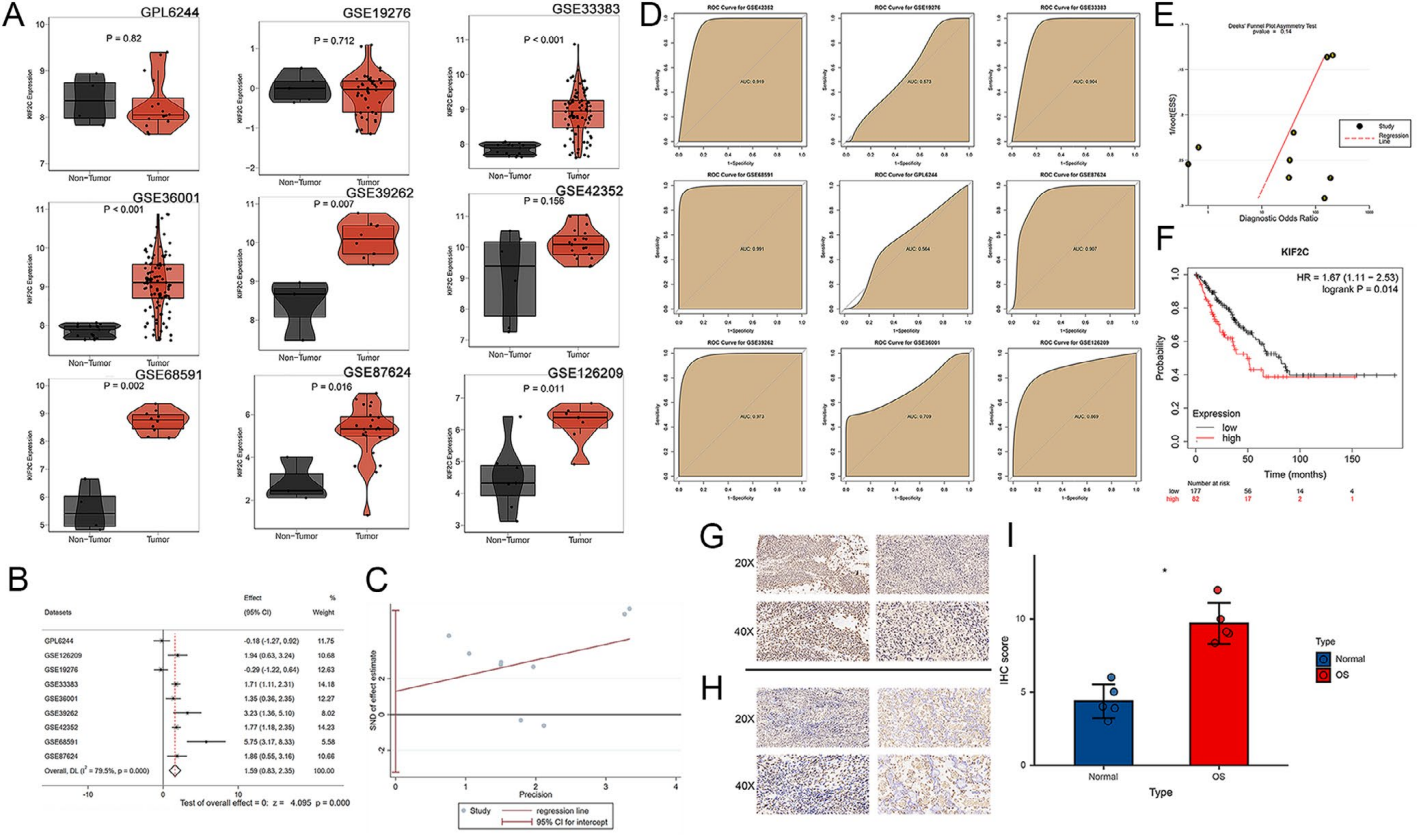
The expression level, diagnostic ability, and clinical significance of KIF2C in OS patients. (A) The differential expression of KIF2C mRNA between OS and normal control samples in 9 datasets. (B) Forest diagram of KIF2C mRNA expression in OS and normal control samples, prompting for high expression. (C) Egger's test showing no publication bias in the analysis of integrating overall OS samples (*p* = 0.519). (D) ROC curves of KIF2C in 9 GEO datasets of OS samples. (E) Deek's funnel diagram, which suggested no publication bias (*p* > 0.05). (F) KIF2C high expression was related to shorter survival time of OS patients, based on the Kaplan Meier Plotter database. (G, H) The expression level of KIF2C protein was higher in OS tissue (A: OS tissues B: Non‐cancer tissues). (I) The bar chart is used for quantifying the scores. KIF2C, Kinesin Family Member 2C; OS, osteosarcoma.

Based on the TCGA TARGET GTEx pan‐cancer dataset, we found KIF2C mRNA expression levels to be significantly upregulated in 33 malignant tumors but downregulated in TGCT (Testicular Germ Cell Tumors) (Figure 1A). The GSEA results showed that the G2M_CHECKPOINT and E2F_TARGETS pathways were highly expressed in all cancers except for STAD, which aligns with the biological role of KIF2C. Additionally, the enrichment of each pathway varied across different cancers, reflecting the regulatory and functional mechanisms of KIF2C in the context of tumor type‐specific backgrounds (Figure 1B). To further investigate its expression within the OS dataset, we first confirmed its role in OS progression using Chronos scores obtained from DepMap (Figure 1C). Subsequently, we gathered nine OS datasets based on predefined inclusion criteria (including dataset merging of GSE11414 and GSE12865, as shown in Figure S1A,B and comprehensive information on the datasets included can be found in Table S1). Our findings indicated that KIF2C expression was upregulated in seven datasets; its expression was not statistically significant in GSE42352. In addition, KIF2C expression levels were downregulated but not statistically significant in GPL6244 and GSE19276 (Figure 2A). Further calculations of SMD and CI for each group indicated that KIF2C expression was upregulated in OS samples (SMD = 1.59, 95% CI [0.83–2.35], *p* < 0.001). Considering the high heterogeneity (*I* ^2^ = 79.5%), a random effects model was chosen (Figure 2B). Egger's and Begg's tests, based on merged expression levels, confirmed no publication bias (Figures 2C and S1C). To investigate the diagnostic potential of KIF2C in OS, ROC curves were plotted and integrated analysis was performed (Figures 2D and S2). The results demonstrated exceptionally high accuracy in distinguishing between OS and NC tissues using KIF2C expression levels, with AUC = 0.94 and 95% CI = 0.92–0.96. These results also showed no publication bias based on the Deek test (Figure 2E). Finally, we utilized the Kaplan–Meier plotter database to determine the prognosis relevance of KIF2C in OS patients and found a close correlation between KIF2C expression levels and poor prognosis (Figure 2F). Finally, IHC results show that KIF2C expression is significantly increased in osteosarcoma tissue, particularly in the perinuclear regions of tumor cells, where staining intensity is notably higher. Some tumor cell areas exhibit stronger staining, suggesting that these cells may be in a more active proliferative state. In contrast, KIF2C expression is lower in healthy bone tissue, with uniform and weak staining, and no significant cell proliferation activity is observed (Figure 2G–I).

### 3.2 KIF2C Expression in Human OS Cell Lines

**FIGURE 3:**
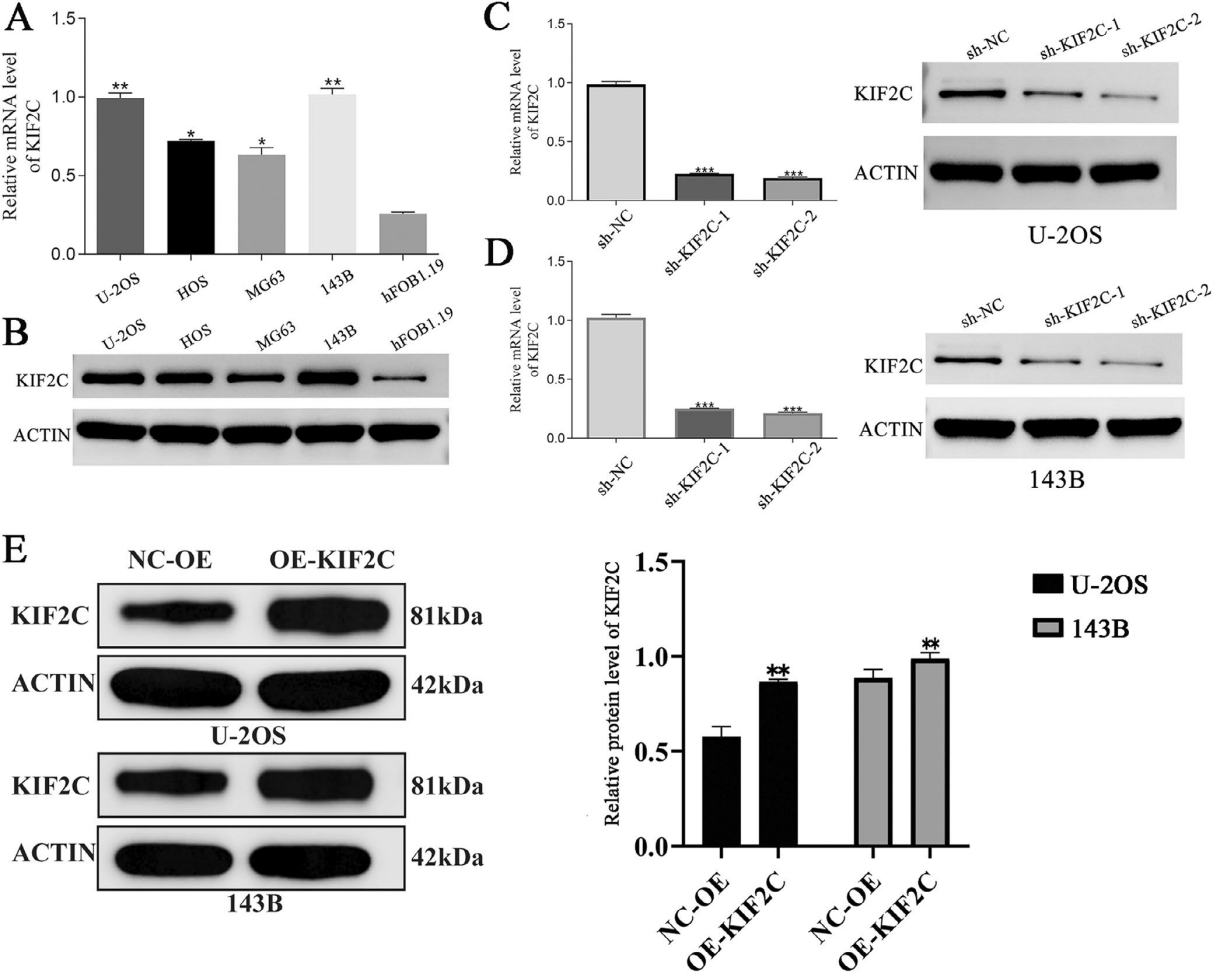
The highly increased expression of KIF2C in OS cell lines. (A, B) RT‐qPCR and WB assay separately determined KIF2C mRNA and protein expression in human OS cell lines (U‐2OS, HOS, MG63, and 143B) was higher than that in human osteoblast cells (hFOB1.19). (C)The determination of KIF2C transfection efficiency in U‐2OS was identified via RT‐qPCR and WB assay. (D)The determination of KIF2C transfection efficiency in 143B was identified via RT‐qPCR and WB assay. (E) Expression of KIF2C protein in U‐2OS and 143B cells after transfection with the KIF2C overexpression plasmid. KIF2C, Kinesin Family Member 2C; OS, osteosarcoma; *p < 0.05; **p < 0.01; ***p < 0.001.

**FIGURE 4:**
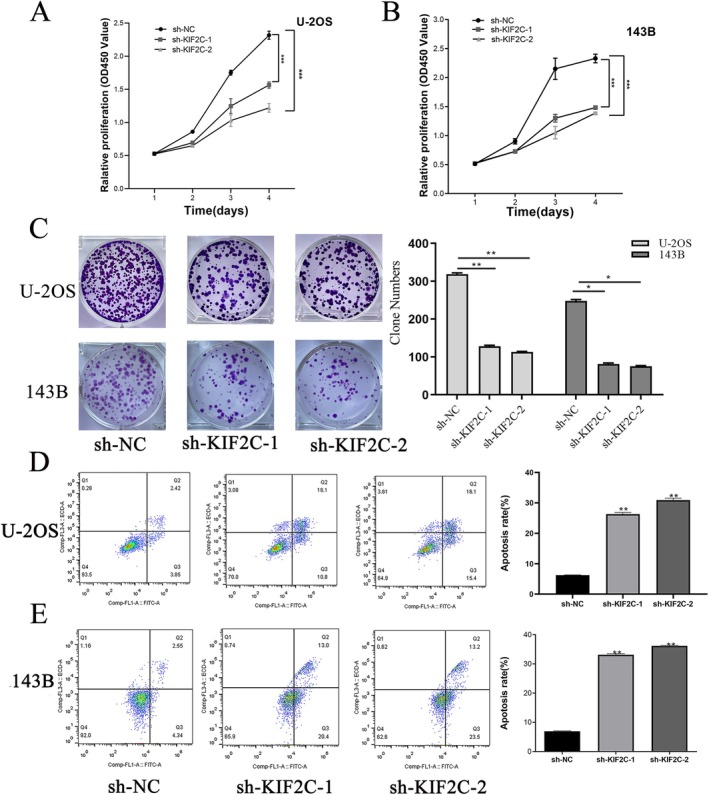
The mediation of KIF2C on proliferation, colony formation, and cell apoptosis in OS. (A) CCK8 assays prompted that KIF2C downregulation inhibited the growth of U‐2OS. (B) CCK8 assays prompted that KIF2C downregulation inhibited the growth of 143B. (C) Downregulating KIF2C impaired colony forming ability in U‐2OS and 143B cell lines. (D, E) Apoptosis assay showed that KIF2C downregulation promoted the apoptosis of the U‐2OS and 143B cell lines, sh‐KIF2C‐2 sites both showed the most prominent apoptosis effect. KIF2C, Kinesin Family Member 2C; OS, osteosarcoma; *p < 0.05; **p < 0.01; ***p < 0.001.

Western blotting and RT‐qPCR were used to assess the expression levels of KIF2C in human OS cell lines. KIF2C had much greater levels of mRNA and protein expression compared to hFOB1.19 cells, particularly in U‐2OS and 143B cells (Figure 3A,B). Therefore, to explore the role of KIF2C in OS, we knocked down KIF2C expression in U‐2OS and 143B cells and compared it with the negative control group (sh‐NC) using RT‐qPCR and Western blotting, while validating transfection efficiency. We found that transfection of plasmids significantly reduced KIF2C mRNA and protein expression levels in U‐2OS and 143B cells, indicating high knockout efficiency (Figure 3C,D). After transfecting U‐2OS and 143B cells with the KIF2C plasmid, KIF2C expression was significantly enhanced in the overexpression group compared to the empty vector control group (Figure 3E). CCK‐8 assay and plate cloning experiments were performed to verify the effects of KIF2C on OS cell proliferation. In comparison with the NC group, KIF2C expression knockdown effectively inhibited cell proliferation and colony formation ability, indicative of the role of KIF2C in promoting OS cell proliferation (Figure 4A–C). Subsequently, flow cytometry was used to investigate the effect of KIF2C on OS cell apoptosis, confirming that decreased expression of KIF2C led to higher U‐2OS and 143B cell apoptosis rates, with the sh‐KIF2C‐2 group showing the highest proportion of apoptotic cells in U‐2OS and 143B cell lines (Figure 4D,E). Above findings indicated that KIF2C inhibits OS cell apoptosis.

### 3.3 Effects of KIF2C on OS Cell Migration, Invasion, and Tumorigenicity

**FIGURE 5:**
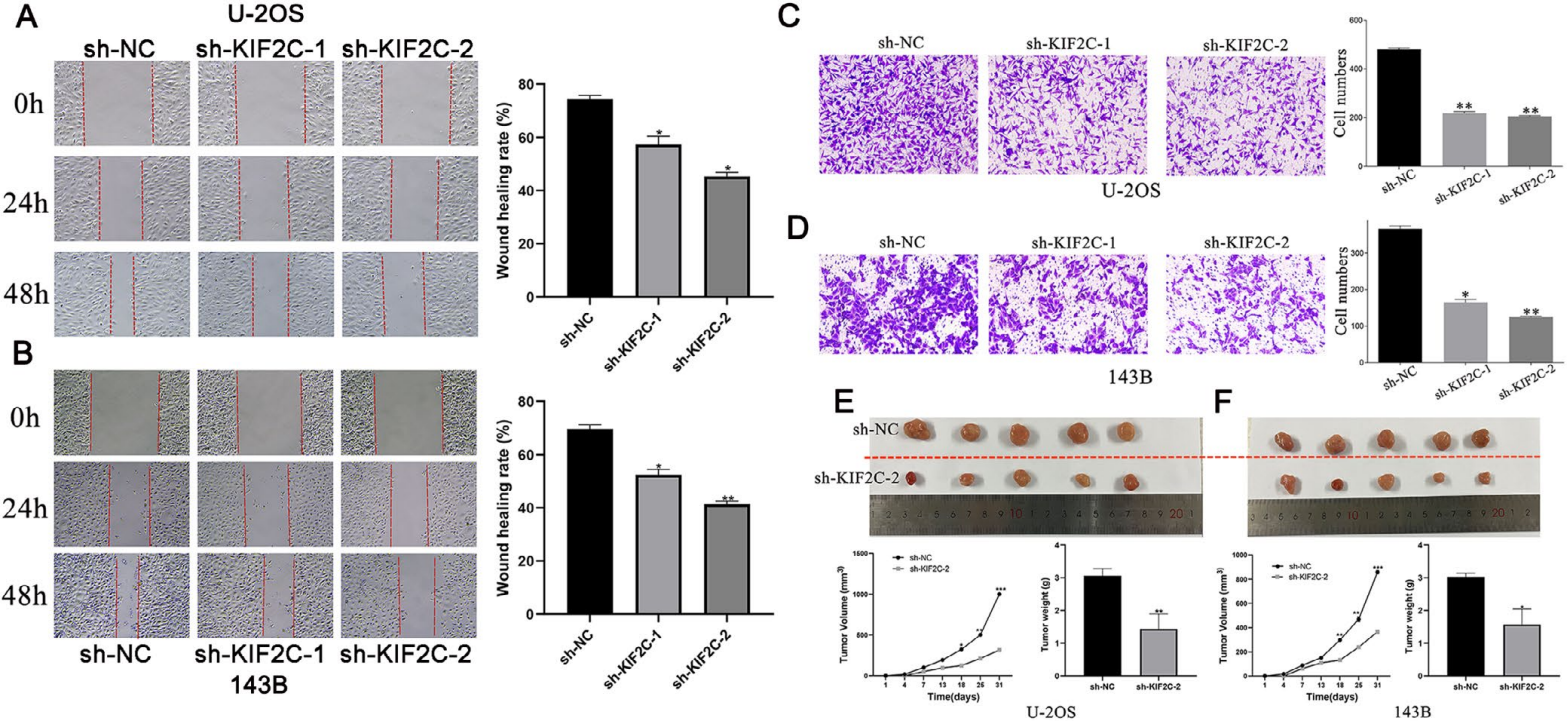
The mediation of KIF2C on migration, invasion, and tumorigenicity in OS. (A, B) After transfecting sh‐KIF2C, the migration ability in U‐2OS and 143B cell lines was both suppressed, which was evaluated by wound‐healing assay. (C, D) Transwell assay was performed to display invasive ability in U‐2OS and 143B cell lines after transfecting sh‐KIF2C. (E, F) The size, volume, and weight of xenograft tumors markedly declined compared with control groups after transfecting sh‐KIF2C‐2. KIF2C, Kinesin Family Member 2C; OS, osteosarcoma; *p < 0.05; **p < 0.01; ***p < 0.001.

In order to uncover more about KIF2C's biological function, we performed wound healing assays and found that KIF2C expression knockdown significantly inhibited the migration ability of U‐2OS and 143B cells (Figure 5A,B). Similar conclusions were drawn from subsequent transwell invasion assays, indicating that targeted inhibition of KIF2C significantly inhibited the invasion of OS cells (Figure 5C,D). Notably, the sh‐KIF2C‐2 site showed the strongest inhibitory effect; therefore, to investigate the consequences of KIF2C on OS cell tumorigenicity in vivo, U‐2OS and 143B OS cells transfected with the stable plasmid sh‐KIF2C‐2 were subcutaneously inoculated in nude mice. The results indicated significantly slower tumor growth in nude mice than in the sh‐NC group (Figure 5E,F). Statistical analysis further revealed that subcutaneous tumor volume and weight in the sh‐KIF2C‐2 group were notably less than that of the sh‐NC group. These results indicated that KIF2C promotes OS cell migration, invasion, and growth in vivo.

### 3.4 KIF2C Overexpression Enhances Malignant Proliferation and Suppresses Apoptosis in Osteosarcoma

**FIGURE 6:**
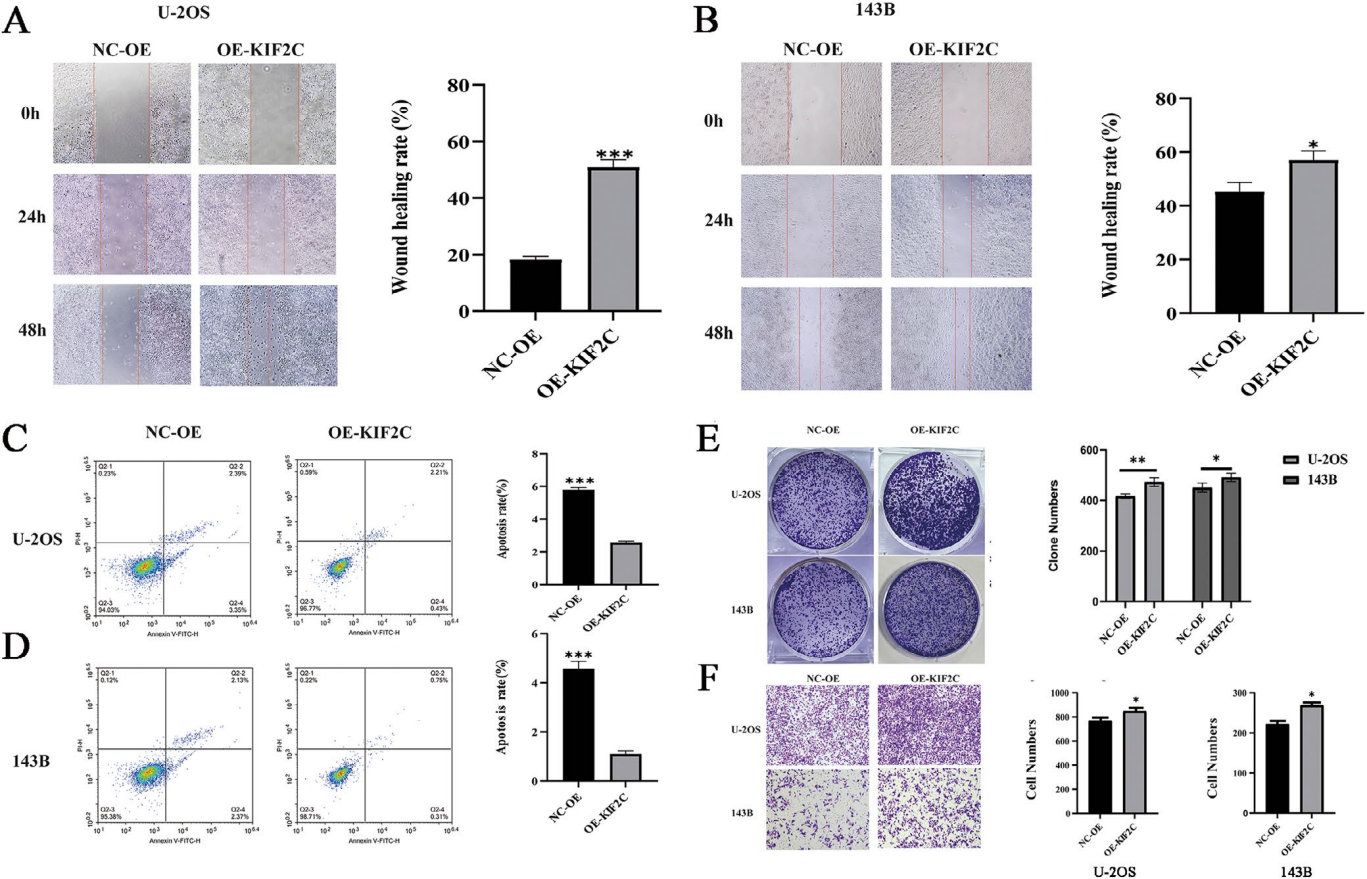
Overexpression of KIF2C promotes malignant proliferation and inhibits apoptosis in osteosarcoma. (A, B) After transfecting with the KIF2C overexpression plasmid, the migration ability in U‐2OS and 143B cell lines was both suppressed, as evaluated by the wound‐healing assay. (C, D) The apoptosis assay showed that KIF2C overexpression suppressed apoptosis in both U‐2OS and 143B cell lines. (E) Overexpression of KIF2C promoted the colony formation ability in U‐2OS and 143B cell lines. (F) Overexpression of KIF2C promoted the invasive ability in U‐2OS and 143B cell lines. KIF2C, Kinesin Family Member 2C; OS, osteosarcoma;  *p < 0.05; **p < 0.01; ***p < 0.001.

Through wound‐healing, apoptosis, colony formation, and Transwell migration assays, we found that overexpression of KIF2C significantly enhanced the invasion ability of osteosarcoma cells. The wound‐healing assay showed that the healing rate of cells with KIF2C overexpression increased, indicating its promotion of cell migration (Figure 6A,B). The apoptosis assay revealed that KIF2C inhibited cell apoptosis, enhancing the survival of tumor cells (Figure 6C,D). The colony formation assay showed an increase in the number of colonies formed by cells with KIF2C overexpression, suggesting its role in promoting cell proliferation (Figure 6E). The Transwell migration assay further confirmed that overexpression of KIF2C enhanced cell migration, supporting its role in invasion and metastasis (Figure 6F). Based on the preliminary research in this study, the above results clearly demonstrate the oncogenic role of KIF2C in osteosarcoma.

### 3.5 Molecular Mechanisms and Action Pathways of KIF2C in OS

**FIGURE 7:**
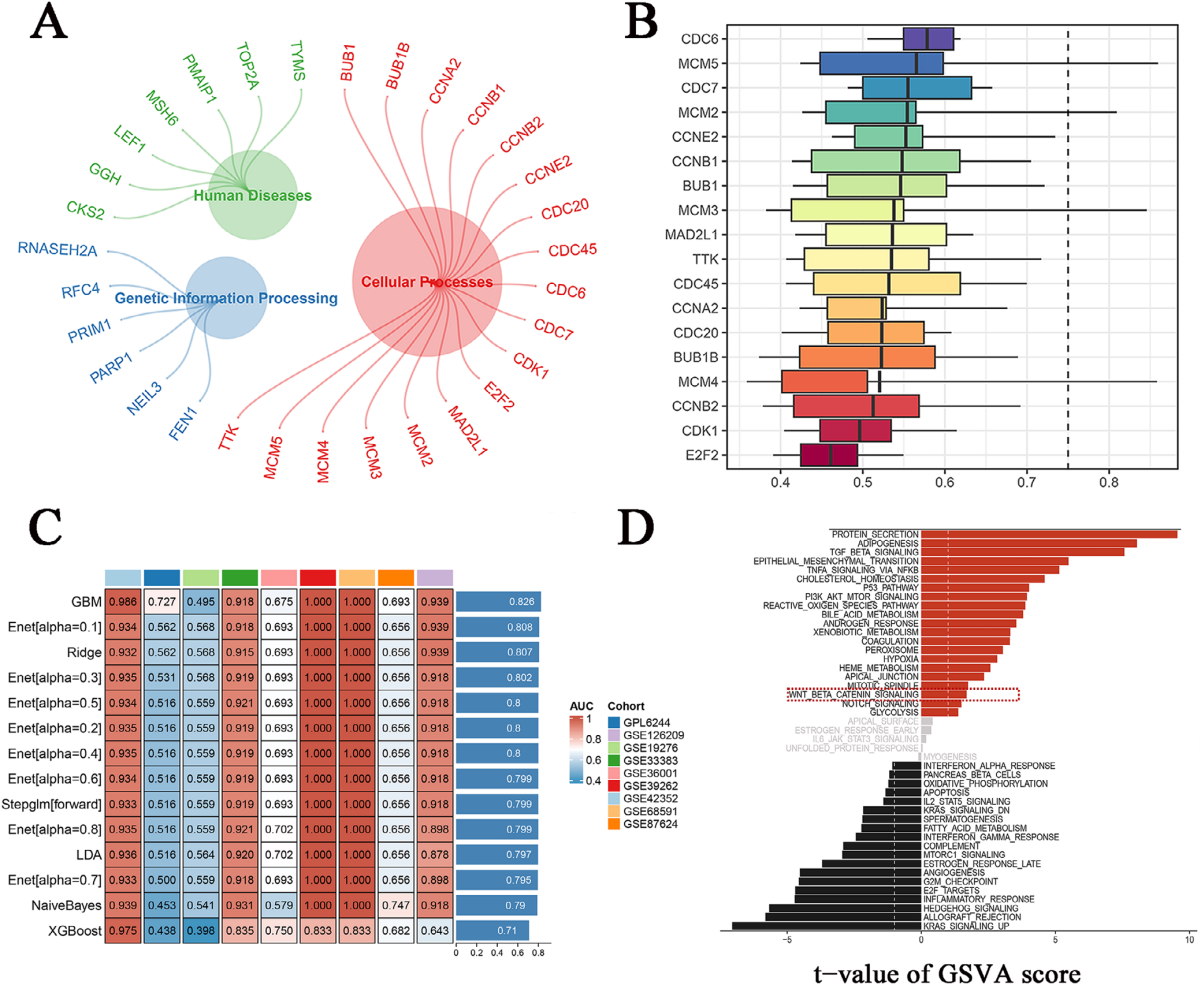
The potential molecular mechanisms of KIF2C in OS. (A) Enrichment terms of KIF2C co‐expressed genes in KEGG pathway. (B) CDC6 has the closest molecular function components to KIF2C, based on GOSemSim algorithm. (C) Classification performance of ensemble machine learning models in identifying KIF2C and CDC6 features. (D) GSVA analysis indicated that high expression of KIF2C is closely related to WNT/β‐catenin pathway. GSVA, Gene Set Variation Analysis; KIF2C, Kinesin Family Member 2C; OS, osteosarcoma.

**FIGURE 8:**
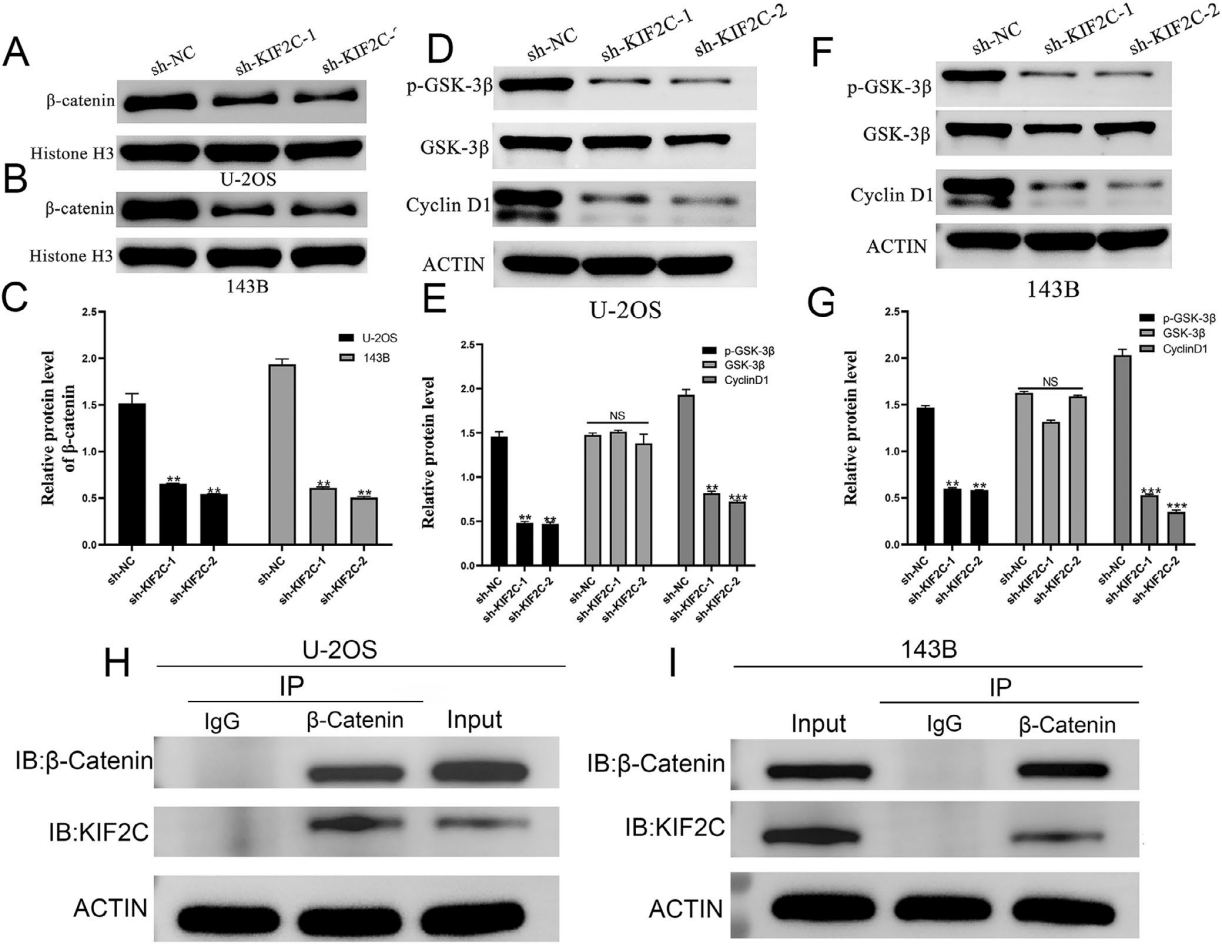
KIF2C regulates the Wnt/β‐Catenin signaling pathway by binding toβ‐Catenin. (A–C) Western blotting was used to unveil the protein level of β‐catenin in the nuclei of U‐2OS and 143B cells. (D, E) The expression of phosphorylated GSK‐3β and Cyclin D1 protein in U‐2OS was reduced in sh‐KIF2C groups, while the GSK‐3β expression level was not statistically different. (F, G) The expression of phosphorylated GSK‐3β and Cyclin D1 protein in 143B was reduced in sh‐KIF2C groups, while the GSK‐3β expression level was not statistically different. (H, I) The interaction between KIF2C and β‐catenin was validated through Co‐IP in U‐2OS and 143B cells. KIF2C, Kinesin Family Member 2C; OS, osteosarcoma; ^ns^ *p* > 0.05; **p < 0.01; ***p < 0.001.

Correlation analysis of the included dataset led to the identification of 1793 KIF2C‐related genes (KIF2C‐RGs) and 146 OS upregulated genes (up‐DEGs). We believe that genes intersecting between KIF2C‐RGs and up‐DEGs, known as KIF2C co‐expression genes, synergize with KIF2C in promoting OS progression (Figure S3). Based on this KIF2C co‐expression gene mining, we explored the molecular mechanisms of KIF2C in OS. KEGG pathway enrichment analysis highlighted cellular processes, genetic information processing, and human diseases as the main enriched pathways. Among these, the cell cycle pathway within cellular processes was the most significant (Figure 7A, Table 1). Subsequent “GO friend” analysis pointed toward CDC6 or genes functionally close to KIF2C in OS [38] (Figure 7B). We observed that by integrating the output probabilities of multiple algorithms, the classification accuracy and stability were significantly improved (Figure 7C). The multivariate logistic regression model, based on logistic regression coefficients and feature expressions, reliably classified each sample (with AUCs greater than 0.7). Notably, in the GBM model, the AUC reached 0.826, which validates that our ensemble machine learning approach effectively captures the underlying biological consensus, providing a solid foundation for further clinical and experimental validation. Furthermore, leveraging GSE42352, we conducted gene set variation analysis by grouping based on the median expression level of KIF2C and performed correlation analysis on corresponding pathway marker genes [51]. The WNT/β‐catenin pathway emerged as having substantial potential (Figures 7D and S4). We then proceeded with a follow‐up study, employing Western blotting to observe changes in β‐catenin expression in OS cell nuclei after KIF2C expression knockdown. Our results revealed a significant reduction in β‐catenin expression in U‐2OS and 143B cell nuclei (Figure 8A–C). Subsequently, we extracted cytoplasmic proteins from U‐2OS and 143B cells and performed Western blotting to assess cyclin D1, GSK‐3β, and phosphorylated GSK‐3β protein expression levels, associated with the cell cycle process. We observed reduced expression of phosphorylated GSK‐3β and cyclin D1 in U‐2OS and 143B cells in the sh‐KIF2C groups, although changes in GSK‐3β expression were not statistically different (Figure 8D–G). These results indicate that the knockdown of KIF2C expression leads to a decrease in β‐catenin levels in the nucleus, thereby inhibiting the expression of the downstream target cyclin D1. Subsequently, the Co‐IP experiment further revealed the interaction between KIF2C and β‐catenin. In U‐2OS and 143B cells, KIF2C protein was detected in the complexes pulled down with β‐catenin antibody, supporting this regulatory effect (Figure 8H,I).

**TABLE 1:** KEGG enrichment analysis based on KIF2C co‐expressed genes in OS samples.

ID	Three‐level classification	*p*	Count	First‐level classification
hsa04110	Cell cycle	8.10E‐20	18	Cellular processes
hsa03030	DNA replication	8.35E‐11	8	Genetic information processing
hsa04114	Oocyte meiosis	2.28E‐06	8	Cellular processes
hsa04914	Progesterone‐mediated oocyte maturation	4.49E‐06	7	Organismal systems
hsa04115	p53 signaling pathway	9.36E‐06	6	Cellular processes
hsa04218	Cellular senescence	1.09E‐04	7	Cellular processes
hsa05166	Human T‐cell leukemia virus 1 infection	7.40E‐04	7	Human diseases
hsa03410	Base excision repair	1.50E‐03	3	Genetic information processing
hsa03430	Mismatch repair	1.09E‐02	2	Genetic information processing
hsa05203	Viral carcinogenesis	1.25E‐02	5	Human diseases
hsa01524	Platinum drug resistance	1.40E‐02	3	Human diseases
hsa01523	Antifolate resistance	1.94E‐02	2	Human diseases
hsa05210	Colorectal cancer	2.17E‐02	3	Human diseases
hsa05222	Small cell lung cancer	2.58E‐02	3	Human diseases
hsa05215	Prostate cancer	2.96E‐02	3	Human diseases
hsa00270	Cysteine and methionine metabolism	4.53E‐02	2	Metabolism

Abbreviations: KIF2C, Kinesin Family Member 2C; OS, osteosarcoma.

### 3.6 Association Between KIF2C and the OS Tumor Microenvironment

**FIGURE 9:**
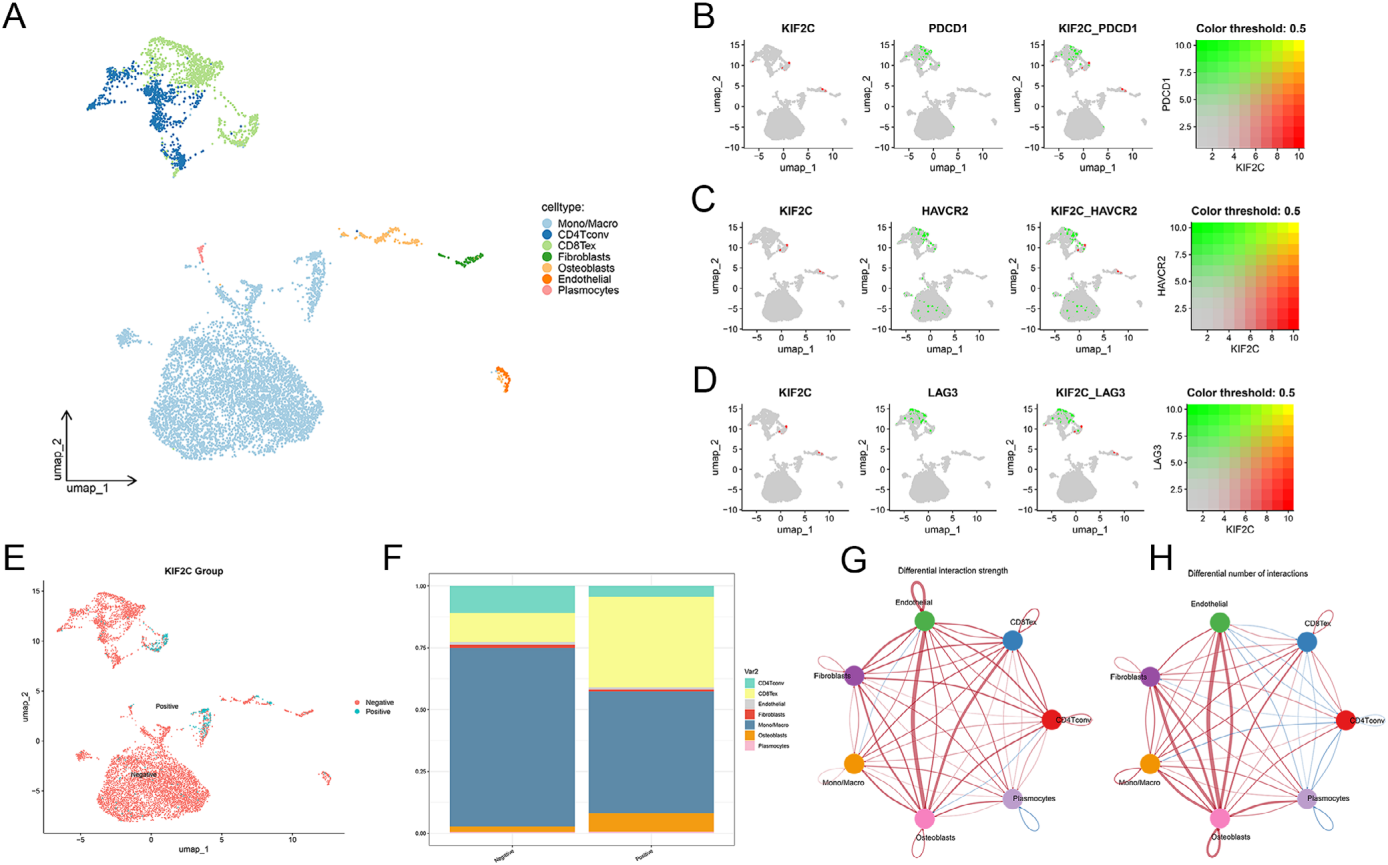
KIF2C is closely associated with immune exhaustion in the osteosarcoma microenvironment. (A) UMAP plot shows the clustering results. (B–D) KIF2C and its correlation with immune exhaustion genes. (E, F) KIF2C expression levels and their relationship with the proportions of different cell subpopulations. (G) The intercellular communication networks in OS by calculating the probability of communications. (H) The intercellular communication networks in OS by calculating the number of communications. KIF2C, Kinesin Family Member 2C; OS, osteosarcoma.

Fibroblasts, immune cells, endothelial cells, and extracellular matrix constituents make up the tumor microenvironment, collectively influencing pathway signal transduction in cancer and immune cells, leading to varied immune responses and epithelial–mesenchymal transition. Besides, the dynamic interplay between these cells is governed by multiple signaling pathways, with the WNT/β‐catenin pathway playing a crucial role in cell migration, cancer stem cell activity regulation, inflammatory response regulation, and immune tolerance [52]. Leveraging GSE42352 and GSE33383 and using the ESTIMATE algorithm, we quantified the association between KIF2C expression levels and immune and stromal scores, as well as tumor purity in OS. We found a close relationship between high KIF2C expression and improved tumor purity in OS, coupled with lower immune and stromal scores (Figure S5A,B). Using the ssGSEA algorithm, we found that higher KIF2C expression correlated with decreased immune cell infiltration levels in the OS tumor microenvironment, resulting in an overall impairment of immune cell function (Figure S6A,B). Subsequent single‐cell sequencing revealed that KIF2C was predominantly expressed in CD8 Tex (i.e., T cell exhaustion) cells (Figure S6C,D), indicative of an association between KIF2C and immune exhaustion in the OS tumor microenvironment. The cell clustering and annotation are shown in Figure 9A. Correlation analysis of KIF2C and CD8 Tex cell markers (PDCD1, HAVCR2, LAG3) revealed a significant positive correlation (Figures 9B–D and S6E–G) [53, 54]. Cells were grouped based on KIF2C expression: KIF2C‐Positive (298 cells) and KIF2C‐Negative (7484 cells), with bar plots showing differences in cell proportions (Figure 9E,F). In the KIF2C‐Positive group, CD8Tex and Osteoblasts increased, while Mono/Macro cells and other immune cells decreased, suggesting KIF2C high expression may promote immune exhaustion and an immunosuppressive microenvironment. Using the CellChat R package, we analyzed cell communication networks to assess interaction strength (Figure 9G) and interaction numbers (Figure 9H). Overall interaction strength increased, but interaction numbers decreased, particularly in CD4+ Tconv cells. We hypothesize that CD4+ Tconv cells in the KIF2C‐Positive group may become hyperactivated, attempting to compensate for cell depletion through stronger signaling, which could exacerbate immune cell dysfunction and advance immune exhaustion. Combined with the results of previous studies indicating that the WNT/β‐catenin pathway can induce CD8 T cell depletion, we believe that KIF2C is closely related to immune exhaustion in the OS tumor microenvironment, a process involving WNT/β‐catenin pathway activation [55, 56].

### 3.7 Potential Small‐Molecule Drugs Targeting KIF2C

**FIGURE 10:**
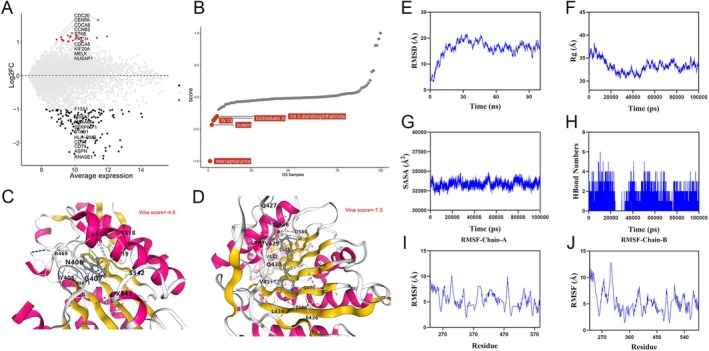
The potential targeting small molecule medicines of KIF2C in OS. (A) Genes with differential expression in OS and normal control samples were displayed using volcano maps. (B) Top five drugs most likely to target KIF2C in primary OS. (C) Molecular docking displayed the affinity between mercaptopurine and KIF2C. (D) Molecular docking displayed the affinity between butein and KIF2C. Molecular dynamics simulation of the protein‐ligand complex. (E) RMSD value of the protein‐ligand complex over time. (F) Rg value of the protein‐ligand complex over time. (G) SASA value of the protein‐ligand complex over time. (H) Number of hydrogen bonds (HBonds) in the protein‐ligand complex over time. (I, J) RMSF value of the amino acid backbone atoms of the protein‐ligand complex over time. KIF2C, Kinesin Family Member 2C; OS, osteosarcoma.

Based on GSE42352, we conducted differential analysis by categorizing KIF2C expression levels and generated a volcano plot (Figure 10A). Subsequently, we utilized the XSum algorithm based on the “PharmacoGx” R package to identify potential small‐molecule drugs targeting KIF2C (Figure 10B). To further validate these findings, we evaluated the affinity between small‐molecule drugs and KIF2C. Butein showed a stronger binding affinity compared to mercaptopurine, with Vina SCOR = −7.0 (Figure 10C,D). Root Mean Square Deviation (RMSD) is an important indicator for assessing the conformational stability of proteins and ligands, reflecting the degree of deviation between atomic positions and the initial position. The smaller the deviation, the better the conformational stability. Therefore, we used RMSD to evaluate the equilibrium of the simulation system. As shown in Figure 10E, the complex system reached equilibrium at 50 ns and fluctuated around 16.2 Å. This indicates that there were slight conformational changes between butein and KIF2C during the simulation. Further analysis revealed that the Radius of Gyration (Rg) and Solvent Accessible Surface Area (SASA) of the complex system exhibited slight fluctuations during the simulation, further confirming that conformational changes occurred in the complex during the motion (Figure 10F,G). Hydrogen bonds play a key role in the binding between ligands and proteins. As shown in Figure 10H, the number of hydrogen bonds between butein and KIF2C during the dynamics process fluctuated between 0 and 6, with the complex typically forming about 3 hydrogen bonds, indicating good hydrogen bond interactions within the complex. Root Mean Square Fluctuation (RMSF) reflects the flexibility of amino acid residues in the protein. As shown in Figure 10I,J, the RMSF values of the complex system showed high flexibility, with the fluctuations of most residues staying below 10 Å. In summary, the complex system underwent slight conformational changes during the simulation. However, overall, the complex binding was relatively stable, with good hydrogen bond interactions. Therefore, butein can achieve a stable binding with KIF2C.

## 4 Discussion

OS, a prevalent malignant tumor in children and adolescents, has an overall survival rate of approximately 20% [57], reflecting a stagnant treatment landscape attributed partly to the complex and unstable genome of OS [58, 59]. In light of this challenge, identifying new biological markers may provide new strategies for OS diagnosis, treatment, and even early prevention. A promising target is KIF2C, encoding a microtubule‐dependent molecular motor crucial for chromosome separation during mitosis. Studies have linked abnormal KIF2C expression to erroneous chromosome aggregation and separation, which can lead to changes in cell genotype and potentially cause uncontrolled cancer cell proliferation [31]. DNA double‐strand breaks reportedly have adverse effects on cell survival and genome stability. In *Xenopus* egg extract and mammalian cells, KIF2C was found to mediate the migration of DNA double‐strand breaks and formation of DNA damage lesions, emerging as a new participant in DNA damage response [7]. In conclusion, the abnormal expression of KIF2C can lead to chromosome structural instability and DNA damage, which may support the close relationship between KIF2C and the occurrence and development of osteosarcoma. However, there is no research to explore the relationship between them.

Herein, our findings revealed significant upregulation of KIF2C mRNA and protein expression levels in patients with OS and in OS cell lines, and demonstrated that OS and NC samples could be differentiated based on KIF2C mRNA levels. Meanwhile, the datasets included in this study were collected from various countries, time points, platforms, and sequencing methods, indicating the potential for substantial heterogeneity and explaining the inconsistencies observed among them. Therefore, we calculated the overall standardized mean difference (SMD) and employed multiple methods to assess heterogeneity, ensuring the credibility and reliability of our conclusions. Subsequently, sensitivity analysis confirmed that the detected heterogeneity did not originate from any single dataset, thereby affirming the robustness of our findings. These results suggested that KIF2C plays an oncogenic role in OS. After determining KIF2C expression levels, we performed various assays to further investigate the role of KIF2C in OS. We found that KIF2C expression knockdown inhibited the proliferation and migration ability of OS cells and promoted their apoptosis. In vivo, reduced KIF2C expression led to a significant reduction in tumor volume and weight in mice, while overexpression of KIF2C promoted malignant proliferation and inhibited apoptosis in OS, highlighting the role of KIF2C in the malignant progression of OS. We thus believe that KIF2C can serve as a biological marker of OS and that targeting KIF2C might be an effective treatment strategy.

In our study, the roles of KIF2C and CDC6 in OS are closely linked, particularly in the regulation of the cell cycle. CDC6 is a crucial factor in the G1/S phase transition, and its inhibition has been shown to significantly reduce OS cell proliferation and invasion [60]. The upregulation of KIF2C may exacerbate this effect by cooperating with CDC6 and other cell cycle regulators, thereby promoting the malignant progression of OS. Specifically, KIF2C may enhance the expression of CDC6 or directly interact with it, thereby affecting the stability of key cell cycle proteins such as Cyclin D1. Cyclin D1 is essential in the G1/S phase transition, and its overexpression can disrupt normal cell cycle regulation, leading to uncontrolled cell proliferation. Moreover, the co‐expression of KIF2C and CDC6 may exacerbate chromosomal instability by disrupting the regulation of chromosome segregation, which plays a critical role in tumorigenesis. The synergistic effect of both proteins may lead to chromosomal mis‐segregation, further contributing to the malignant transformation of OS cells.

Through GSVA analysis, we identified a significant association between KIF2C and the WNT/β‐catenin pathway. Knockdown of KIF2C led to a marked decrease in the nuclear expression of β‐catenin, accompanied by the suppression of Cyclin D1 expression. CO‐IP experiments further confirmed a direct interaction between KIF2C and β‐catenin. Given KIF2C's microtubule‐depolymerizing activity, we hypothesize that KIF2C may regulate the WNT/β‐catenin pathway through two potential mechanisms: on one hand, KIF2C might influence the nucleocytoplasmic distribution of β‐catenin by modulating microtubule dynamics, as studies have shown that the microtubule network plays a crucial role in β‐catenin nuclear transport [61]; on the other hand, the direct binding of KIF2C to β‐catenin could stabilize the β‐catenin/TCF transcriptional complex, enhancing its ability to activate downstream target genes (e.g., Cyclin D1, c‐Myc), a mechanism analogous to the functions of other kinesin family members such as KIF14 [62]. Furthermore, the association between KIF2C and LEF1 expression levels was analyzed based on multiple datasets. LEF1, a transcription factor, serves as a nuclear effector in the WNT/β‐catenin pathway [51]. It plays a central role as a mediator of WNT/β‐catenin signaling and downstream cellular effects, and it also participates in stem cell maintenance. The positive correlation between KIF2C and LEF1 suggests that KIF2C may further amplify WNT signaling by regulating LEF1‐mediated transcriptional activity, thereby contributing to the maintenance of stem‐like properties in OS cells. Therefore, KIF2C may act as an upstream regulator of the WNT/β‐catenin pathway through direct binding and microtubule‐dependent regulation, promoting the malignant progression of OS. Future studies should further explore the specific molecular mechanisms underlying the relationship between KIF2C, microtubule dynamics, and β‐catenin nuclear transport, as well as its interaction with LEF1, providing deeper theoretical insights for KIF2C‐targeted OS therapies.

Leveraging the ssGSEA and ESTIMATE algorithms, we observed that high KIF2C expression was associated with reduced infiltration levels of immune and stromal cells, suggesting an immune‐depleted tumor microenvironment. Single‐cell sequencing data further revealed that KIF2C expression was highest in CD8+ exhausted T (Tex) cells and showed a significant positive correlation with Tex cell marker genes (PDCD1, HAVCR2, LAG3). This finding aligns with the established concept that chronic antigen stimulation in the tumor microenvironment drives T cells into an exhausted state, characterized by progressive functional impairment and elevated expression of inhibitory receptors such as PD‐1, TIM‐3, and LAG‐3. These receptors not only dampen T cell effector functions but also contribute to resistance against immune checkpoint inhibitors. The association between KIF2C and Tex cell markers raises the possibility that KIF2C may play a role in promoting or maintaining T cell exhaustion, potentially through its interaction with the WNT/β‐catenin pathway, which has been implicated in immune evasion and Tex cell development. Future studies should investigate whether KIF2C directly modulates Tex cell differentiation or function, and whether targeting KIF2C could reverse T cell exhaustion and enhance the efficacy of immunotherapy in osteosarcoma [63].

Our prediction of potential small‐molecule drugs targeting KIF2C led to the identification of butein as a promising candidate. Butein has demonstrated apoptosis‐inducing effects on OS cells by regulating oxidative stress, activating the JNK signaling pathway, and blocking the Akt/mTOR signaling pathway [64]. These findings provided us with a certain theoretical basis for our research. Butein, a plant polyphenol, exhibits potential for alleviating bone cancer pain [65]. We believe that the combination of butein and methotrexate–doxorubicin–cisplatin offers a new perspective for improving OS cure rates.

This study has some limitations, primarily stemming from the small sample size within the Gene Expression Omnibus database. Considering the high heterogeneity of results, multicenter clinical experiments and extensive large‐scale prospective studies need to be conducted. In addition, the predicted small‐molecule drug butein, identified through molecular docking and further characterized by molecular dynamics simulations, has not yet been experimentally validated in osteosarcoma models. While the computational analyses provided robust insights into the binding stability and dynamics between butein and KIF2C, in vitro and in vivo experiments are ultimately required to confirm its therapeutic efficacy and mechanism of action. Future studies should prioritize the experimental validation of butein's effects on osteosarcoma cells, including its ability to inhibit KIF2C activity and its impact on tumor growth and metastasis.

## 5 Conclusion

To summarize, through computational biology and in vitro and in vivo experiments, we confirmed that KIF2C functions as an oncogene in OS and regulates the WNT/β‐catenin pathway to promote the malignant progression of OS. Moreover, KIF2C is involved in OS cell proliferation, migration, invasion, and tumorigenicity in vivo. In terms of molecular mechanisms, KIF2C promotes OS progression by regulating the WNT/β‐catenin pathway. Further, it is closely related to immune exhaustion in the OS tumor microenvironment. We believe that high KIF2C expression can robustly diagnose OS and is linked to worse prognosis of patients with OS. Targeting KIF2C may offer new therapeutic approaches in managing OS.

## Author Contributions

The authors' contributions are as follows: Y.‐Y.L. contributed to conceptualization, funding acquisition, resources, and writing the original draft. W.S. was involved in software development and writing the original draft. L.L. performed formal analysis and data curation. J.‐H.C. and J.‐T.L. contributed to validation and visualization. Z.‐T.H. and M.O. participated in writing – review and editing, as well as validation.

## Ethics Statement

Our study was completed in accordance with the Declaration of Helsinki and approved by the ethics Committee of the Jiangxi provincial People's Hospital (Jiangxi, China); mouse studies were carried out at The First Affiliated Hospital of Nanchang Medical College (20212BAB206059 and NYLLSC20240411). All experiments were performed in accordance with relevant guidelines and regulations.

## Conflicts of Interest

The authors declare no conflicts of interest.

## Supporting information


Figure S1.

Table S1.

## Data Availability

The datasets generated and analyzed during the current study are available in the Therapeutically Applicable Research to Generate Effective Treatments (TARGET) database (www.ocg.cancer.gov/programs/target); The Gene Expression Omnibus (GEO, www.ncbi.nlm.nih.gov/geo/) database; TCGA pan‐cancer dataset in the UCSC Xena database (http://xena.ucsc.edu/); PubChem database (https://pubchem.ncbi.nlm.nih.gov/); PDB (http://www.rcsb.org/pdb/home/home.do); AutoDock Vina 1.2.2 (http://autodock.scripps.edu/).
