# Supplementary material for: Elevated KIF2C Expression Drives Osteosarcoma Progression by Modulating the Wnt/β‐Catenin Signaling Pathway and Contributing to an Immunosuppressive Tumor Microenvironment
Source: Cancer Med. 2025 Apr 28;14(9):e70915. doi: 10.1002/cam4.70915 (PMC12035763; doi:10.1002/cam4.70915)
Supplement: Supplementary file 1 — Figure S1. [file CAM4-14-e70915-s002.docx]

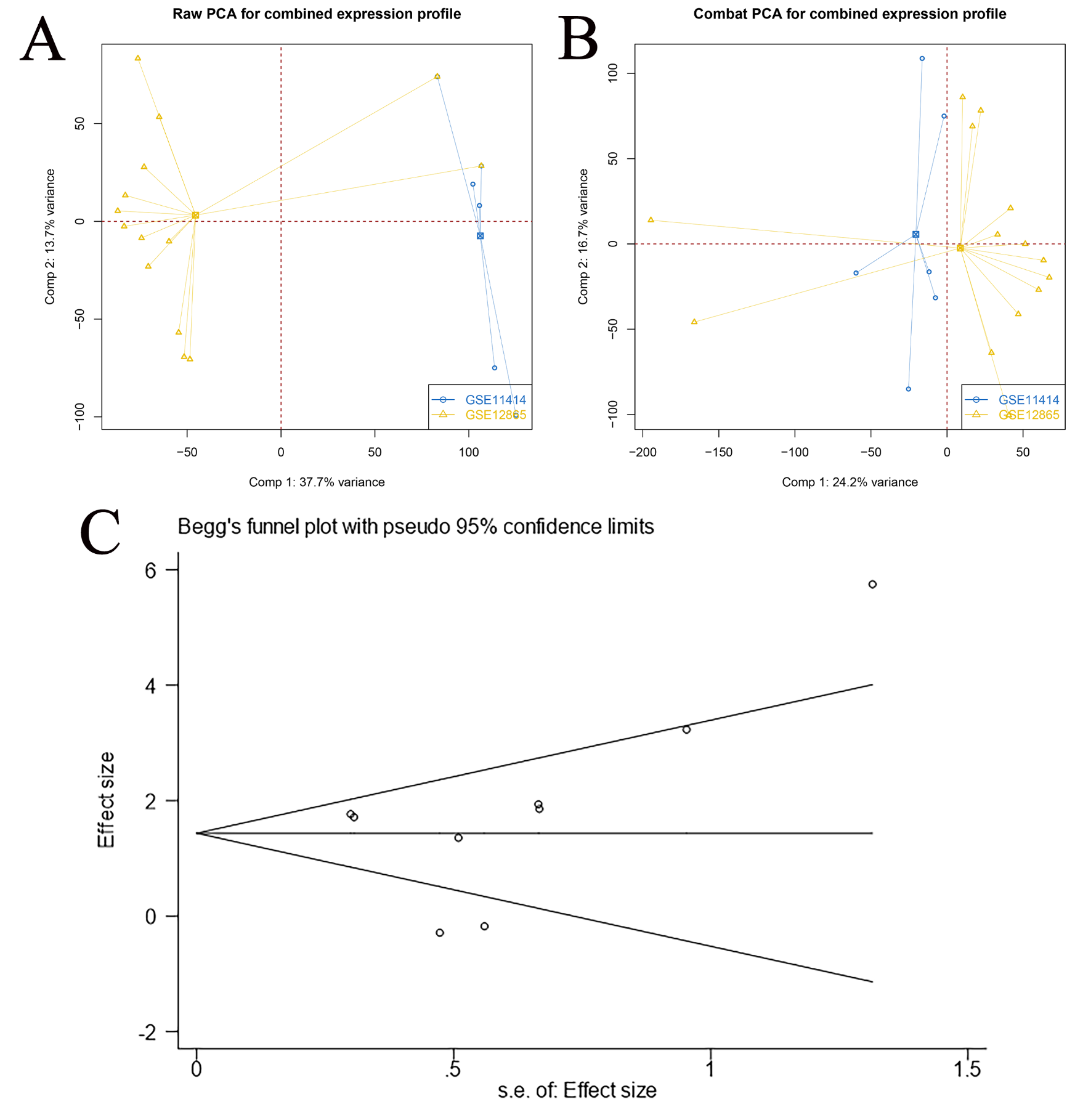


**Supplementary Fig. 1.** (A) PCA dimensionality reduction before datasets merging (GSE11414 and GSE12865). (B) PCA dimensionality reduction after datasets merging (GSE11414 and GSE12865). (C) Begg’s test showing no publication bias in the analysis of integrating OS samples (*p*=0.348).


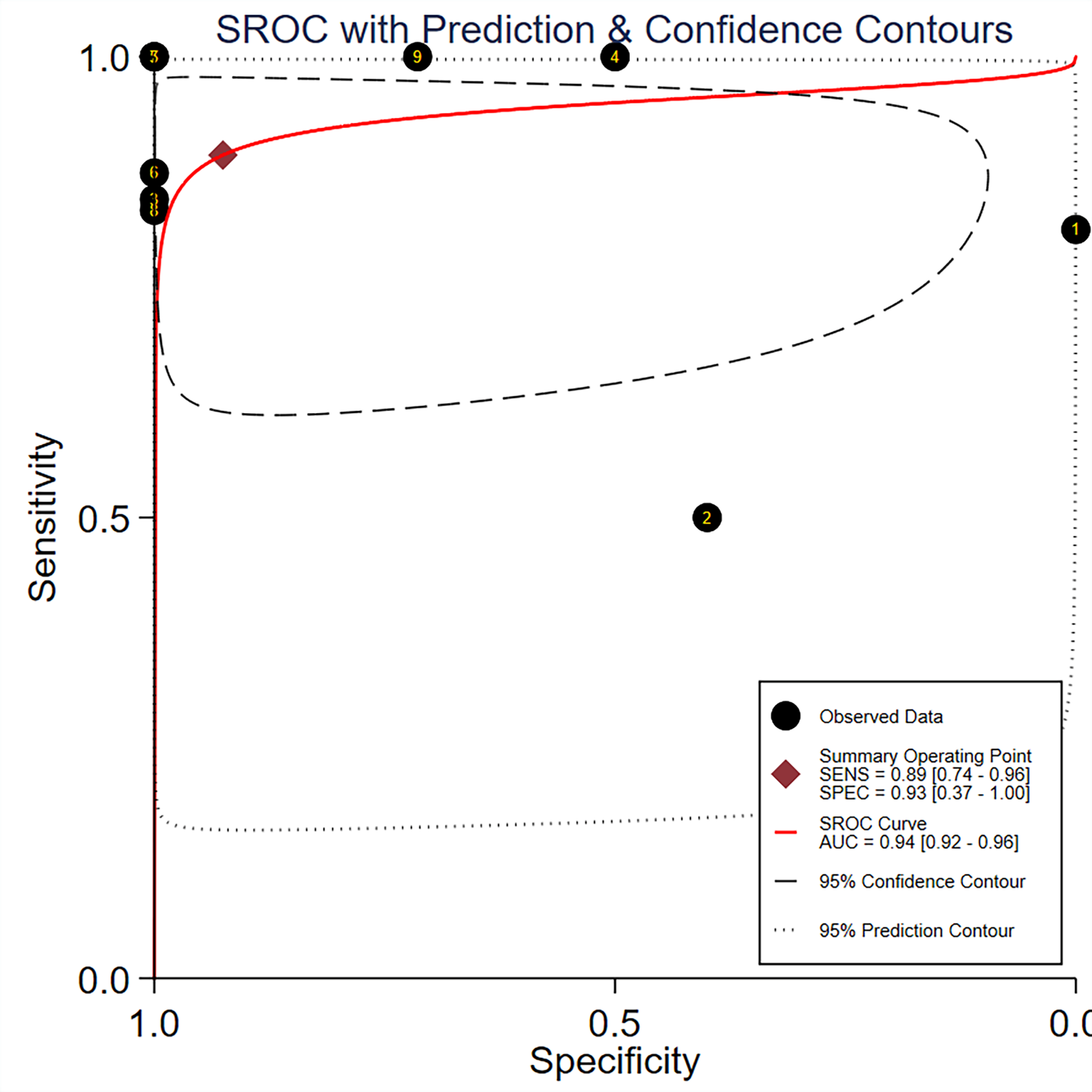


**Supplementary Fig. 2.** The sROC with prediction and confidence contours in OS samples and normal control samples, AUC=0.94.


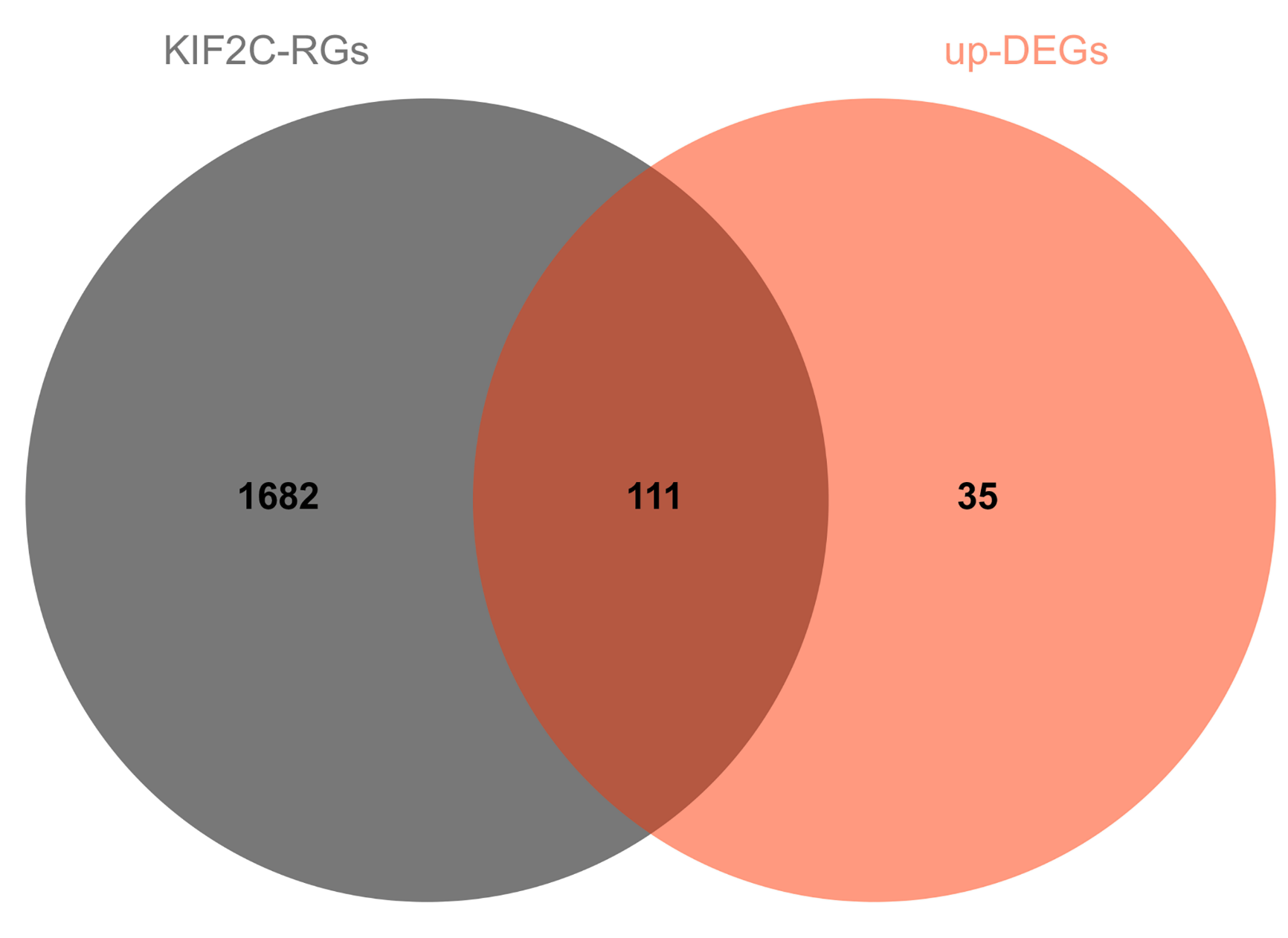


**Supplementary Fig. 3.** Venn diagram showing the KIF2C co‐expressed genes in OS based on up‐DEGs and KIF2C‐RGs.


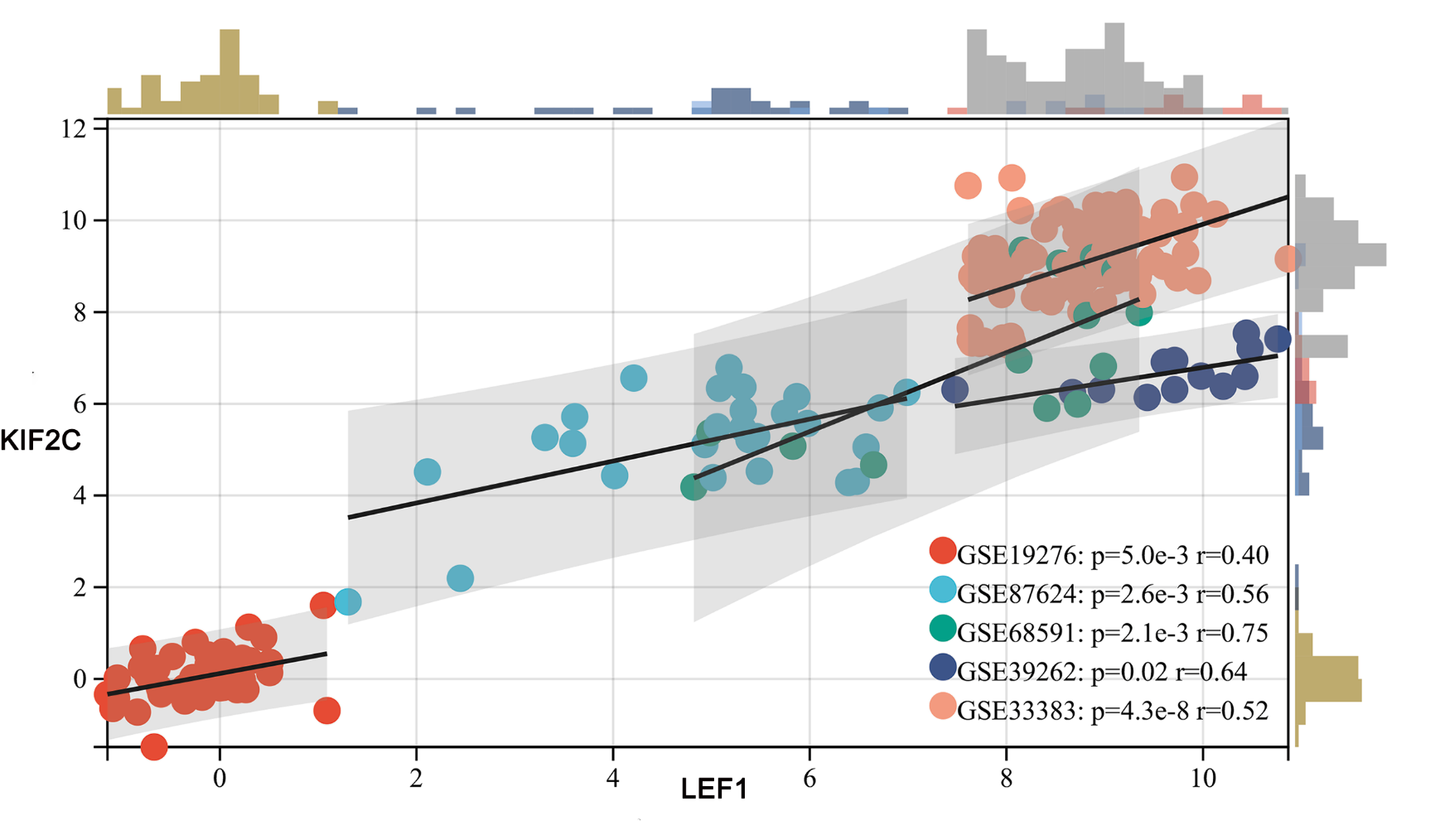


**Supplementary Fig. 4.** The correlation analysis between KIF2C and LEF1 in multiple datasets.


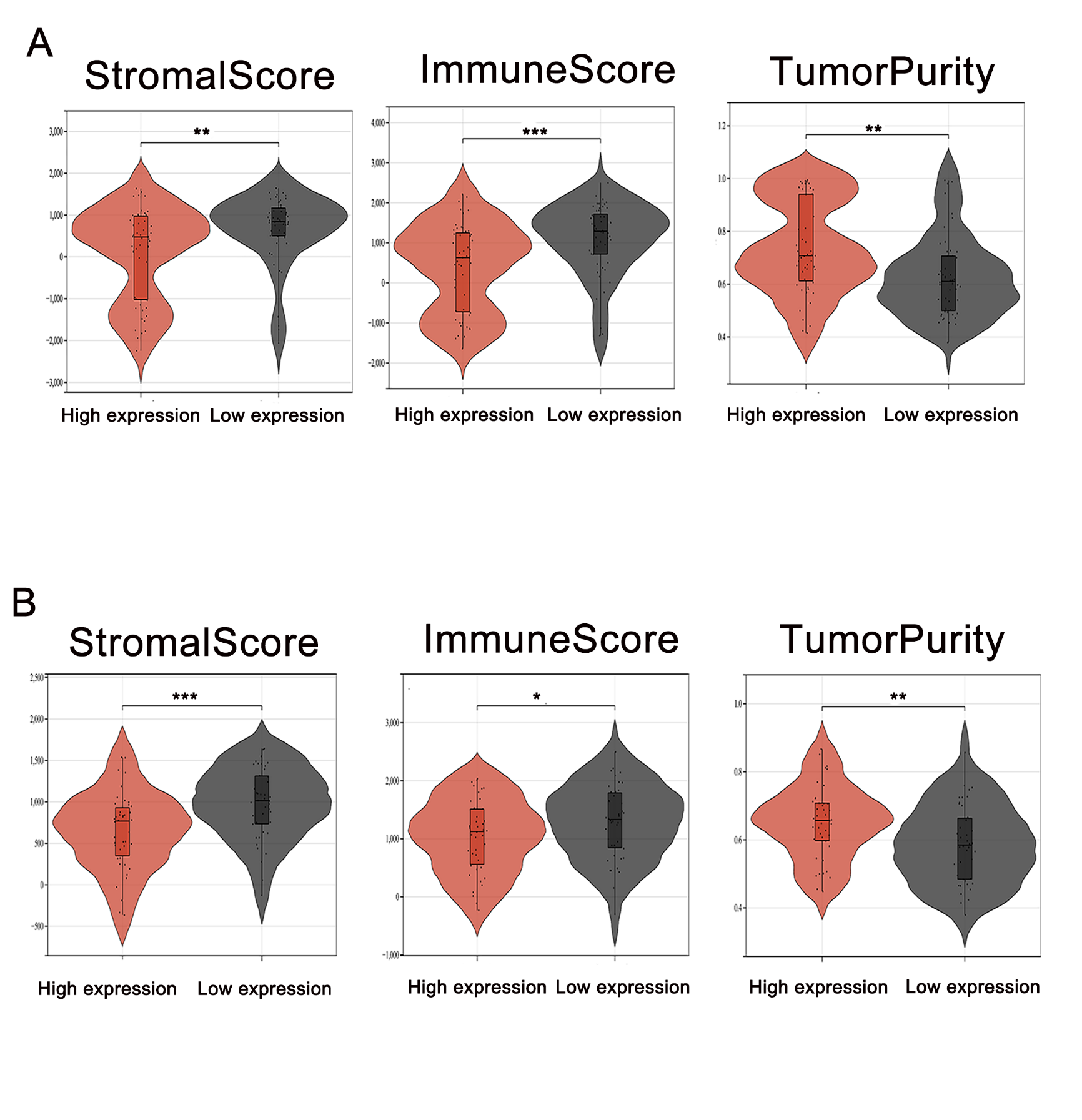


**Supplementary Fig. 5.** (A) The ESTIMATE algorithm showed that high expression of KIF2C was closely related to the improvement of tumor purity in OS and was associated with lower immune and stromal scores in GSE42352. (B) The ESTIMATE algorithm showed that high expression of KIF2C was closely related to the improvement of tumor purity in OS and was associated with lower immune and stromal scores in GSE33383.


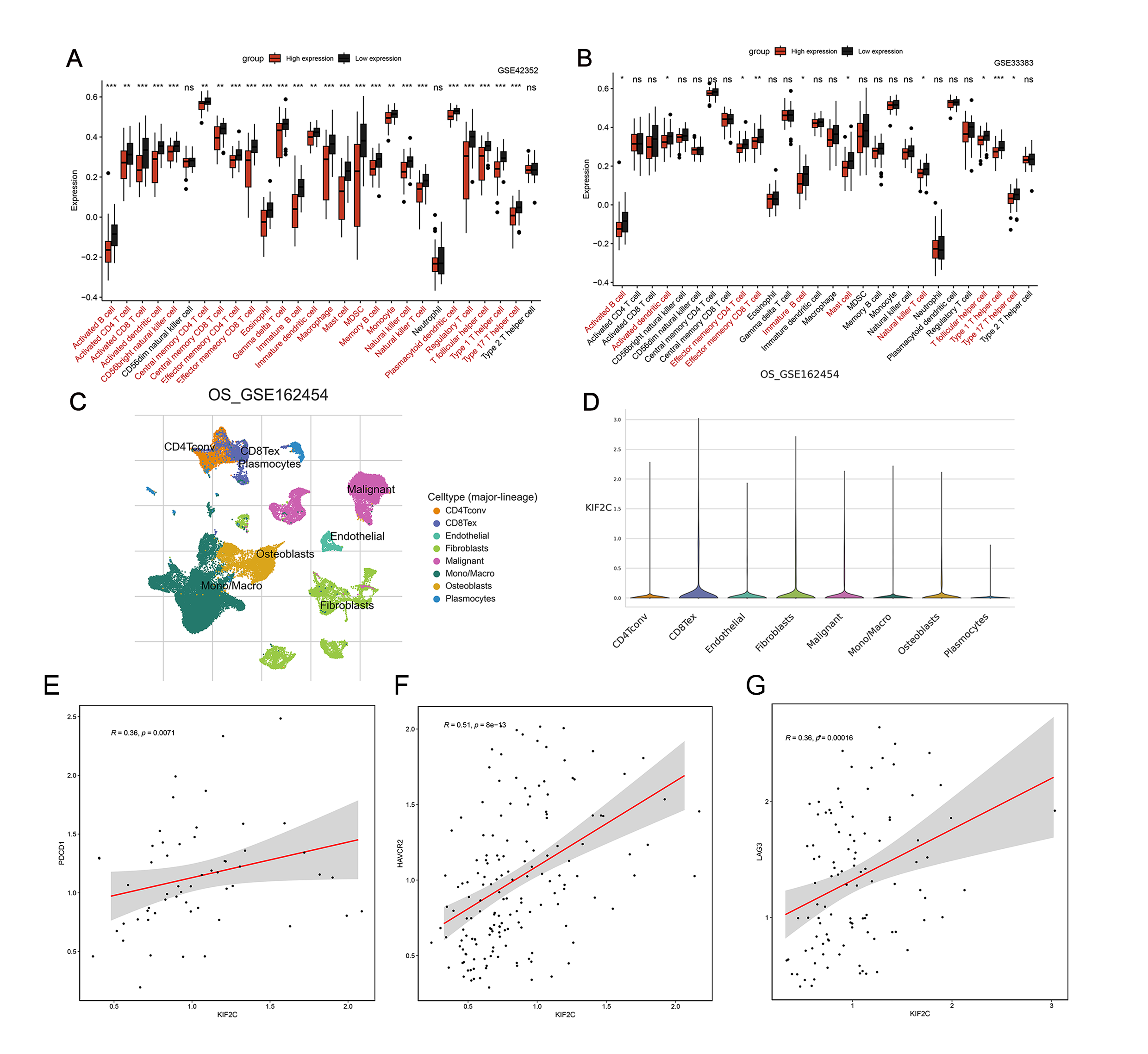


**Supplementary Fig. 6 The effect of KIF2C on tumor microenvironment of OS** (A-B) Reling on the ssGSEA algorithm, KIF2C overexpression was associated with a decrease in the infiltration level of multiple immune cells in OS in GSE42652 and GSE33383 datasets. (C-D) Different cell types of OS microenvironment based on the TISH2 database was shown, while CD8Tex cells have the highest level of expression among them. (E-G) The correlation between KIF2C and CD8 Tex cell marker genes PDCD1, HAVCR2, LAG3 at the single-cell leve.
