# Supplementary material for: Elevated KIF2C Expression Drives Osteosarcoma Progression by Modulating the Wnt/β‐Catenin Signaling Pathway and Contributing to an Immunosuppressive Tumor Microenvironment
Source: Cancer Med. 2025 Apr 28;14(9):e70915. doi: 10.1002/cam4.70915 (PMC12035763; doi:10.1002/cam4.70915)
Supplement: Supplementary file 2 — Table S1. [file CAM4-14-e70915-s001.docx]

Supplementary Table 1: Details of the public datasets included in this study.

| Datasets | Country | Year | Platform | Samples number | Data type |
| --- | --- | --- | --- | --- | --- |
| GSE11414 | Canada | 2008 | GPL6244 | 6 | Microarray |
| GSE12865 | Canada | 2008 | GPL6244 | 14 | Microarray |
| GSE19276 | Australia | 2009 | GPL6848 | 49 | Microarray |
| GSE33383 | Norway | 2011 | GPL10295 | 99 | Microarray |
| GSE36001 | Norway | 2012 | GPL6102 | 25 | Microarray |
| GSE39262 | United Kingdom | 2012 | GPL96 | 13 | Microarray |
| GSE42352 | Norway | 2012 | GPL10295 | 118 | Microarray |
| GSE68591 | USA | 2015 | GPL11028 | 12 | Microarray |
| GSE87624 | USA | 2016 | GPL11154 | 47 | RNA-seq |
| GSE126209 | China | 2019 | GPL20301 | 11 | RNA-seq |
